# Color‐map recommendation for MR relaxometry maps

**DOI:** 10.1002/mrm.30290

**Published:** 2024-10-16

**Authors:** Miha Fuderer, Barbara Wichtmann, Fabio Crameri, Nandita M. de Souza, Bettina Baeßler, Vikas Gulani, Meiyun Wang, Dirk Poot, Ruud de Boer, Matt Cashmore, Kathryn E. Keenan, Dan Ma, Carolin Pirkl, Nico Sollmann, Sebastian Weingärtner, Stefano Mandija, Xavier Golay

**Affiliations:** ^1^ Radiotherapy, Division Imaging and Oncology University Medical Center Utrecht Utrecht The Netherlands; ^2^ Department of Diagnostic and Interventional Radiology University Hospital Bonn Bonn Germany; ^3^ Department of Neuroradiology University Hospital Bonn Bonn Germany; ^4^ Undertone.design Bern Switzerland; ^5^ The Institute of Cancer Research and Royal Marsden Hospital London UK; ^6^ Department of Diagnostic and Interventional Radiology University Hospital Wuerzburg Wuerzburg Germany; ^7^ Department of Radiology University of Michigan Ann Arbor Michigan USA; ^8^ Department of Medical Imaging Henan Provincial People's Hospital & the People's Hospital of Zhengzhou University Zhengzhou China; ^9^ Laboratory of Brain Science and Brain‐Like Intelligence Technology Biomedical Research Institute, Henan Academy of Sciences Zhengzhou China; ^10^ Department of Radiology and Nuclear Medicine Erasmus MC Rotterdam The Netherlands; ^11^ Philips MR Clinical Science Eindhoven The Netherlands; ^12^ National Physical Laboratory Teddington UK; ^13^ Physical Measurement Laboratory National Institute of Standards and Technology Boulder Colorado USA; ^14^ Department of Biomedical Engineering Case Western Reserve University Cleveland Ohio USA; ^15^ GE HealthCare Munich Germany; ^16^ Department of Diagnostic and Interventional Radiology University Hospital Ulm Ulm Germany; ^17^ Department of Diagnostic and Interventional Neuroradiology, School of Medicine, Klinikum rechts der Isar Technical University of Munich Munich Germany; ^18^ TUM‐Neuroimaging Center, Klinikum rechts der Isar Technical University of Munich Munich Germany; ^19^ Department of Imaging Physics Delft University of Technology Delft The Netherlands; ^20^ Queen Square Institute of Neurology University College London London UK; ^21^ Gold Standard Phantoms Sheffield UK; ^22^ Bioxydyn Manchester UK

**Keywords:** color, display, quantitative MR, relaxation, standardization

## Abstract

**Purpose:**

To harmonize the use of color for MR relaxometry maps and therefore recommend the use of specific color‐maps for representing $T_{1}$, $T_{2}$, and $T_{2}^{*}$ maps and their inverses.

**Methods:**

Perceptually linearized color‐maps were chosen to have similar color settings as those proposed by Griswold et al. in 2018. A Delphi process, polling the opinion of a panel of 81 experts, was used to generate consensus on the suitability of these maps.

**Results:**

Consensus was reached on the suitability of the logarithm‐processed Lipari color‐map for $T_{1}$ and the logarithm‐processed Navia color‐map for $T_{2}$ and $T_{2}^{*}$. There was consensus on color bars being mandatory and on the use of a specific value indicating “invalidity.” There was no consensus on whether the ranges should be fixed per anatomy.

**Conclusion:**

The authors recommend the use of the logarithm‐processed Lipari color‐map for displaying quantitative $T_{1}$ maps and $R_{1}$ maps; likewise, the authors recommend the logarithm‐processed Navia color‐map for displaying $T_{2}$, $T_{2}^{*}$, $R_{2}$, and $R_{2}^{*}$ maps.

This work originated with the Quantitative MR Study Group of the International Society of Magnetic Resonance in Medicine (ISMRM); it has the approval of the Publication Committee and of the Board of the ISMRM.

## 1 INTRODUCTION

MRI for clinical applications commonly uses qualitative $T_{1}$‐weighted and $T_{2}$‐weighted images, complemented by a plethora of other images based on different contrast mechanisms. Traditionally, these images are displayed in grayscale. Yet, MRI can also deliver quantifiable physical parameters including the relaxation time constants $T_{1}$ and $T_{2}$ and their inverses ($R_{1}$ and $R_{2}$), as well as related quantitifiable entities like $T_{2}^{*}$, $R_{2}^{*}$, $T_{2}^{\prime }$, and $R_{2}^{\prime }$. Mapping/imaging of these quantitative parameters dates back to at least the 1970s^1^ but only gained traction in the last two decades, after acquisition techniques for quantification were optimized.^2^, ^3^, ^4^, ^5^ In the recent decade, this has included many types of transient‐state sequences.^6^, ^7^, ^8^, ^9^, ^10^

In many clinical areas,^1^, ^11^ quantitative relaxation maps have shown benefits over more conventionally used weighted images; an overview of clinical/translational applications is provided in a review by Tippareddy et al.^12^ There are also applications in which a quantitative map is an intermediate product intended for synthesis (i.e., simulation) of “weighted” images.^13^ In addition, qualitative images are very sensitive to imaging parameters leading to strong variations among systems and vendors; these variations often impede generalization of machine‐learning algorithms trained on one vendor's data to other imaging systems.^14^ Quantitative maps, by their nature, could solve this issue, provided the measurements are sufficiently reproducible.^15^

Typically, quantitative maps (most notably, quantitative relaxation maps) are displayed in *color*. The use of color coding was first described by Pykett et al. for displaying the $T_{1}\left(x,y,z\right)$ distribution, which in turn provides a visual representation with greater contrast visibility than grayscale images.^1^, ^11^ A *color‐map* defines the way in which way a scalar quantity is to be displayed in color. It is a function that maps the scalar values of a range (e.g., from 0% to 100%, or from 0 to 255 for 8 bits of storage, or from 20 ms to 50 ms) onto a path through a (3D) color space. With this definition, there is an infinite variety of possible color‐maps. This paper focuses on quantitative relaxation maps. A *proton density* map, which is often a by‐product of generating relaxation maps, is not handled here; in current practice, the predominant color‐map to display proton density maps is grayscale (see Supporting Information Data S1). However, there is no consensus yet on how to display relaxation maps.

**FIGURE 1:**
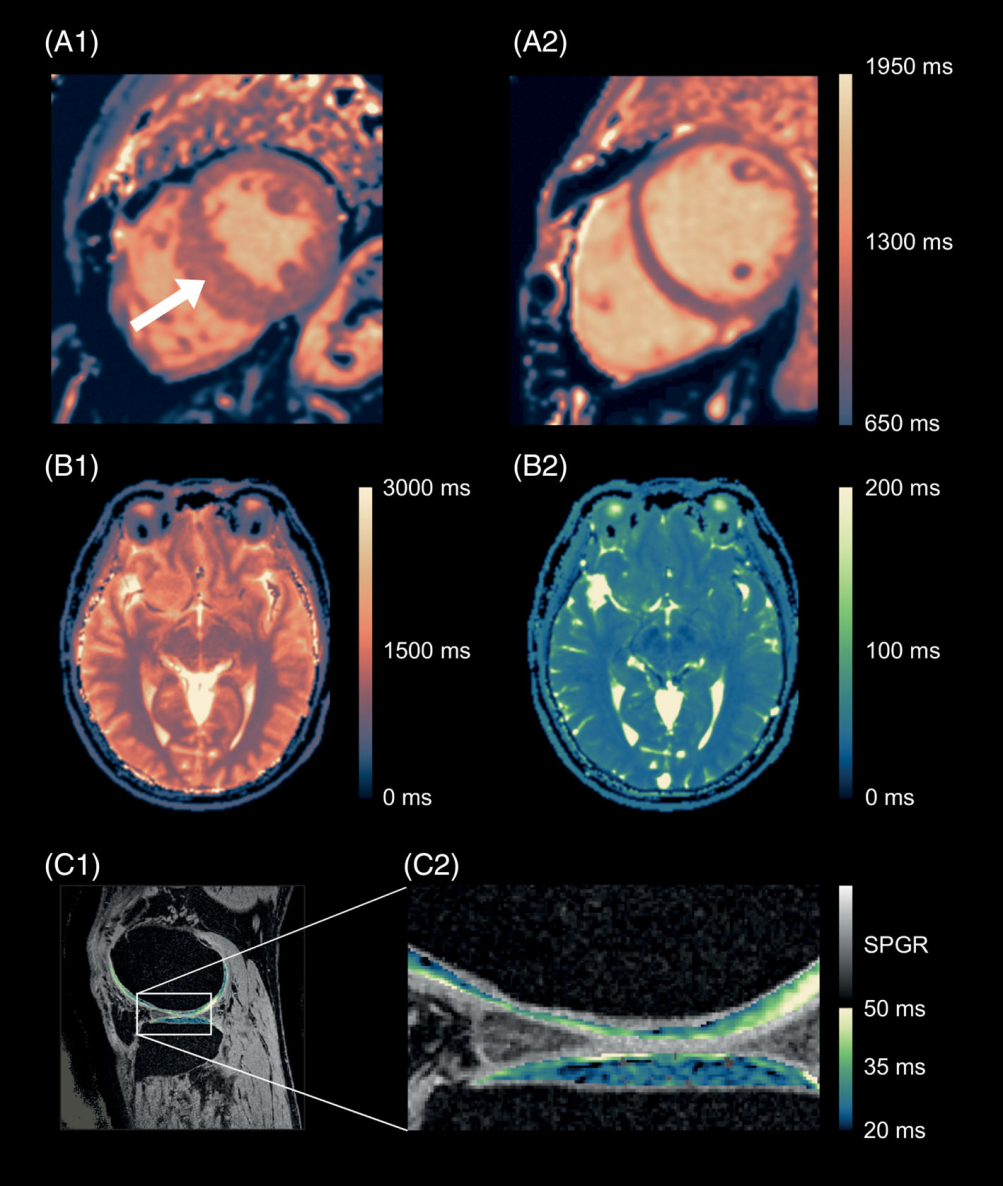
(A1,A2) Maps of the myocardium of 2 different patients; healthy tissue (A2) may be depicted as purplish, whereas an orange‐like color points to pathology (A1; *white arrow*). (B1) Example of a brain T_1_ map. (B2) Example of a brain T_2_ map. (C1) Example of an image providing a T_2_ map of a knee (see C2 for detail); it contains a T_2_ map of cartilage (*in color*) as well as the context of “weighted” anatomy information (*in gray*).

In quantitative imaging, there are several reasons for displaying images in color rather than in grayscale. First, a color is easier to remember, to compare, and to communicate than a grayscale level (Figure 1A1,A2). Second, by systematically using one color‐map for $T_{1}$ maps (Figure 1B1) and another, a very different color‐map for $T_{2}$ maps (Figure 1B2), the viewer can immediately recognize the image as a $T_{1}$ map, a $T_{2}$ map, or, alternatively, as a qualitative (“weighted”) grayscale image. This characteristic is particularly relevant if one combines several types of images into one displayed image, as in Figure 1C: The color part displays a $T_{2}$ map of the cartilage, whereas the grayscale‐displayed regions represent an image with mixed $T_{1}$/$T_{2}$ weighting, providing the anatomical context. The color immediately indicates the image type ($T_{2}$ map or anatomical context), even within one image. Furthermore, as observed by Pykett et al., for a given range, a color‐map allows for more contrast visibility than a grayscale image, allowing for better distinction between normal and diseased tissue.

Many of these benefits (e.g., comparing, communicating, recognizing) can only be achieved if a standardized system of color‐maps is used. Unfortunately, there exists a large variety of applied color‐maps in recent literature on quantitative MR (Supporting Information Data S2). In addition, many of the applied color‐maps do not adhere to scientific standards, as postulated in the field of data visualization^16^ as “perceptually uniform, perceptually ordered, color‐vision deficiency and color‐blind friendly, and readable in black and white prints.”

Perceptual uniformity is particularly relevant for the ability to distinguish between normal versus abnormal tissue: Because we are dealing with many potential anatomies and pathologies, and many potential range settings, the disease‐critical threshold could be in any portion of the range. Perceptual uniformity ensures that each portion of the displayed range shows sufficient contrast visibility.

To arrive at a community‐driven consensus, the Delphi method (see Section 2) has previously been successful in a variety of fields. It has been initially applied for military purposes^17^ and as a structured tool to forecast the (technological) future.^18^ In the medical field, the Delphi method has been applied for a variety of purposes^19^, ^20^, ^21^, ^22^, ^23^, ^24^ and more specifically in imaging applications, like the recommendation of processes for renal MRI,^25^, ^26^, ^27^ MRI/CT/ultrasound of small bowel and colon,^28^ prostate cancer MRI,^29^ and for lesion segmentation.^30^

To recommend and promote a well‐motivated choice for the use of color‐maps for the display of quantitative MR relaxation parameter maps, earlier work of Griswold et al.^31^ was used as a starting point. A Delphi process was conducted to achieve a consensus recommendation for clinical adoption. The aim of the study was to provide distinct recommendations for color‐maps designed for quantitative MRI relaxometry applications to be used in research as well as in the clinic.

## 2 METHODS

This effort originated with the Quantitative MR Study Group (qMR‐SG) of the International Society of Magnetic Resonance in Medicine (ISMRM), initiated by the first author (M.F.) and supported by members of the qMR‐SG board (X.G., B.B., D.M., M.C.). This group of five people recruited experts in the field of quantitative MR, using the following criteria: including both clinical and technical expertise, as well as experts on color‐mapping, experts on the process, and industry representatives, while striving for a spread over geography, gender, and, particularly, balancing physicists and clinicians. This process led to a group of initially 16 people, the Color Recommendation Committee (CRC), which is composed of the authors of this publication plus one representative of Canon and one representative of Philips, replacing Ruud de Boer after his retirement.

A Delphi process was used to develop a consensus recommendation for the display of color‐maps. This process relied on a panel of experts who answer questionnaires in two or more rounds. After each round, a facilitator provided an anonymized summary of the experts' forecasts from the previous round as well as the reasons they provided for their judgments. Experts were encouraged to revise their earlier answers in light of the replies of other members of their panel.

### 2.1 Defining the panel

A panel of 50–100 representative experts, which is central to the Delphi process, was selected as follows:

- Selection of 25 of the 100+ responders to the original email—an informal email to the qMR‐SG members in February 2022, polling the idea of a joint effort toward standardization of color‐maps for relaxation. The selection was based on those responders who actively contributed to a discussion on the need to standardize color‐maps (25 addressees).
- A random pick of 17 other respondents to the aforementioned email discussion.
- Key experts recommended by the CRC members (20 addressees).
- Addition of 1 scientist on MRI, known to be color‐blind (1 addressee).
- Request of societies to suggest panel members, to obtain a better balance over anatomies (8 addressees):
  ∘ Quantitative Imaging Biomarkers Alliance
  ∘ European Association of Cardiovascular Imaging
  ∘ European Society of Radiology
  ∘ Society of Abdominal Radiology
  ∘ European Society of Gastrointestinal and Abdominal Radiology
  ∘ Society for Advanced Body Imaging.
- Another round of suggestions by CRC members, specifically aimed at including more clinicians (9 addressees).
- All CRC members were part of the panel and answered the questionnaires (16 addressees).

Of the 96 addressees, 81 expressed willingness to cooperate (including the one person known to be color‐blind); these 81 were defined as the panel.

Panel demographics were collected in Delphi Round 2 (out of 4).

### 2.2 Definition of questionnaires

A 9‐point Likert scale was used in most questions, spanning the range from “fully disagree” to “fully agree.” In general, the questionnaires went from generic to specific. Round 1 started with generic questions like establishing the number of required color‐maps: Should these be anatomy‐specific? Should they differ between $T_{1}$ and $T_{2}$? (See Table 1 for the list of questions.) Furthermore, the relative importance of features like availability and perceptual linearity was probed.

**TABLE 1:** Results from Round 1.

Question	Score	Consensus?
(Number of respondents: 58 out of 81)		
One and the same color‐map for T_1_, for example, should be used for all anatomies.	78% agreed	Yes
Within a given anatomy, it is a good idea to use a single color‐map for all relaxation properties, including T_1_, T_2_, $T_{2}^{*}$, T_2_', T_1_rho, R_1_, R_2_. So a T_1_ map may have a similar look to, e.g., a T_2_ map.	59% disagreed	No
Within a given anatomy, it is a good idea to define *exactly two* color‐maps (i.e., one for all longitudinal‐magnetization related relaxation [T_1_, R_1_] and one color‐map for all of T_1_, T_2_, $T_{2}^{*}$, T_2_', T_1_rho, R_2_ and R_1_rho). (If a relaxation rate map, such as R_1_, uses the reverse color‐map of a relaxation time map, such as T_1_, this does not count as a different color‐map.)	41% disagreed	No
Within a given anatomy, we need *more than two* different color‐maps (e.g., one for each relaxation property) to represent all possible relaxation‐related information.	45% agreed	No
The range of an applied color‐map should in no way be fixed to certain relaxation values but should always be freely adaptable.	43% agreed	No
A color‐map should contain a specific color (e.g., black), clearly distinguishable from all the other colors of the color‐map, to indicate invalidity (e.g., for regions of the image containing no nuclei and therefore having no, or unknown, relaxation properties).	90% agreed	Yes
Most (or all?) MR images are presented with a dark background.	57% agreed	No
The maps shown in Griswold 2018 should serve as a basis for the recommended color‐maps.	38% agreed	No
The proposed color‐maps should be as perceptually linear (Crameri 2020) as possible.	7.7 ± 1.4	Yes, important
The proposed color‐maps should be as perceptually linear as possible also when viewed by people with deuteranopia (most common red/green blindness; see Crameri 2020).	6.9 ± 1.8	Yes, fairly important
The proposed color‐maps should be as perceptually linear as possible also when viewed by people with any kind of color blindness.	6.2 ± 2.1	No*
The proposed color‐maps should be as perceptually linear as possible also when converted to grayscale.	6.7 ± 2.3	No*
The proposed color‐maps should be available on common processing platforms like *Pyplot* and *Matplotlib*.	7.5 ± 2.3	No*
The proposed color‐maps should be available for free.	8.1 ± 1.0	Yes, very important

*Note*: For the first eight items, agreement was reached when either agreement or disagreement exceeded 75%. Only the category coming closest to consensus is provided, which may be either the Likert categories 1, 2, and 3 or the Likert categories 7, 8, and 9. For the last six questions, asking about relative importance, the criterion was σ<2.0.

* These items did not reach consensus, because the SD exceeded 2.0.

**FIGURE 2:**
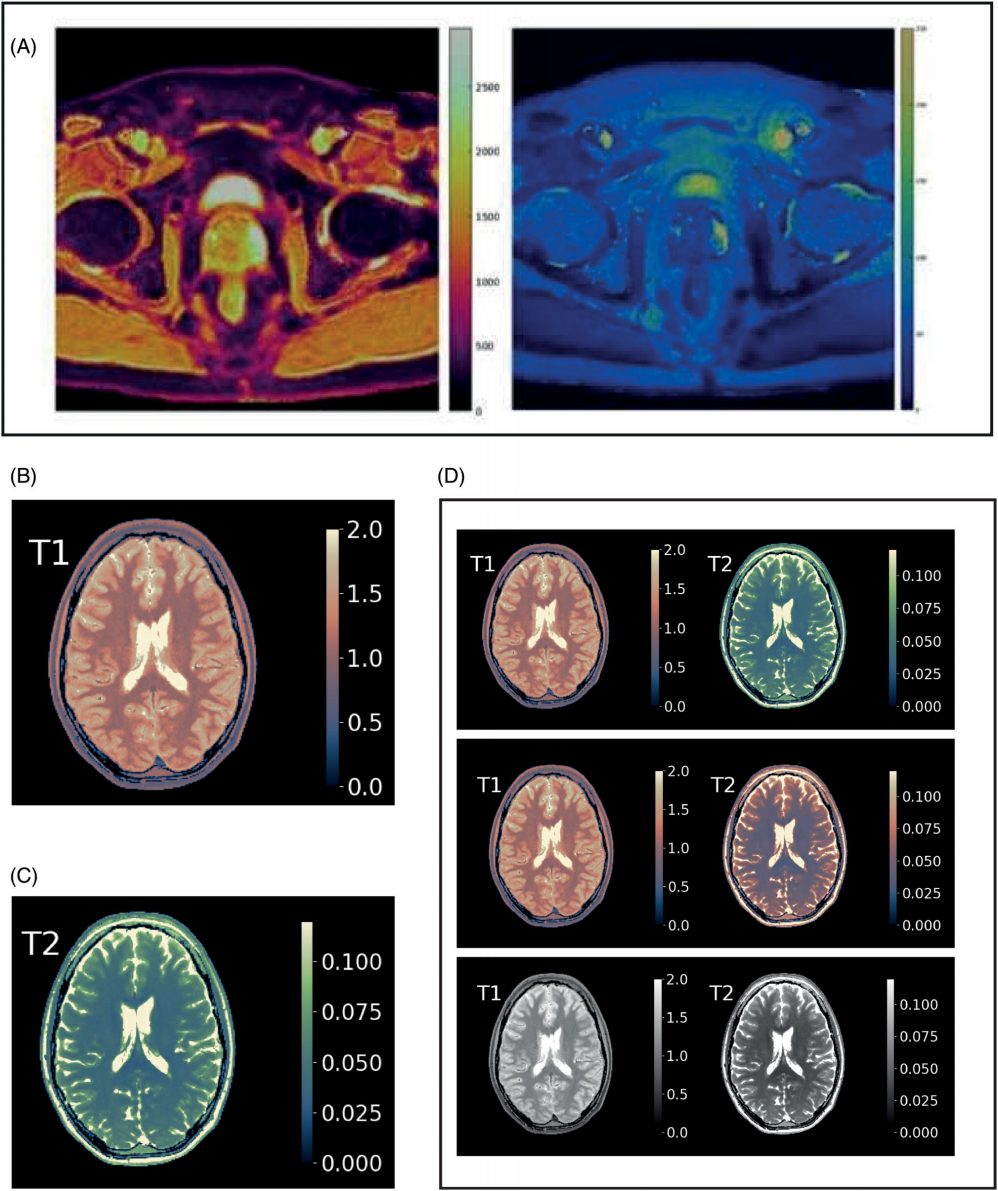
Images shown in Rounds 1 and 3 of the Delphi process. (A) Example of Griswold 2018 color‐maps (copied from ref. 31). (B) Initial version of the Lipari map. (C) Initial version of the Navia map. (D) The (visualized) multiple‐choice question of Round 3: “Lipari for $T_{1}$, Navia for $T_{2}$”↔ “Lipari for both $T_{1}$ and $T_{2}$” ↔ “Gray for both $T_{1}$ and $T_{2}$.”

We asked one question specifically about the color‐maps by Griswold et al.,^31^ which was motivated by the fact that Griswold's color‐maps strive for the same aim as outlined in the present recommendation (perceptually linear, with two distinct color‐maps for $T_{1}$ and $T_{2}$). So, we attempted a shortcut by asking one's opinion to the statement, “The maps shown in Griswold 2018 should serve as a basis for the recommended color‐maps,” showing the image set of Figure 2A as an example. If this were to result in a clear agreement, then this would define the recommendation on color‐maps.

In response to the comments of earlier rounds, some questions were added, reformulated, grouped, or split in subsequent rounds. For instance, in Round 2, the questions on the number of maps were reformulated. The question in the statement, “The range (…) should be fixed,” was split into “clinical” and “scientific” (see Table 2). Furthermore, the original three questions on perceptual linearity for different types of color‐blindness were collapsed into one single question.

**TABLE 2:** Results from Round 2.

Question	Score	Consensus?
(Number of respondents: 48)		
T_1_ maps and T_2_ maps should get the same color‐map.	(a)	No
T_2_ maps and $T_{2}^{*}$ maps should get the same color‐map.	58% agreed	No
T_2_ maps and T_1_rho maps should get the same color‐map.	44% agreed	No
Next to color‐maps for T_2_ and T_1_rho map(s), there should be (an) additional color‐map for T_2_‐dispersion and/or T_1_rho‐dispersion.	(b)	No
R_1_ maps should get the same color‐map as T_1_ maps, or the inverse thereof.	79% agreed	Yes
For clinical work, the range of an applied color‐map should not be fixed to certain relaxation values but should always be freely adaptable.	(a)	No
For clinical work, it is useful to have recommendations (per type of map and possibly per anatomy and field strength) on the range to be applied.	72% agreed	No
For scientific work (e.g., on the efficacy of obtaining quantitative maps), the range of an applied color‐map should not be fixed to certain relaxation values but should always be freely adaptable.	57% agreed	No
In current clinical practice, quantitative relaxation MR images are always read with dark background.	79% agreed	Yes
The maps shown in Griswold 2018 should serve as a basis for the recommended color‐maps.	47% agreed	No
The proposed color‐maps should be as perceptually linear as possible and when viewed by people with any kind of color blindness (i.e., perceptually linear when considering only the luminance component).	7.1 ± 1.6	Yes, important

*Note*: On the “T_1_ maps and T_2_ maps should get the same color‐map” statement, 54% disagreed, 38% agreed, and there was almost no middle ground. The same was true for the question on freely adaptable ranges. On dispersion, 40% of panel members scored a “5,” which was interpreted as predominantly “no opinion.”

As an example of a comment‐prompted question, Round 3 contained the question on the statement, “Each quantitative relaxation image must be displayed in conjunction with a color‐bar with adequately readable numbers.”

As of Round 3, initial versions of the Lipari and Navia maps were presented (see Figure 2B,C), although the question was still open on whether $T_{1}$ and $T_{2}$ should get different maps (Figure 2D). The Lipari and Navia were not presented as competing against the Griswold 2018 maps, but as a refinement thereof, particularly on perceptual uniformity for all types of vision.

**FIGURE 3:**
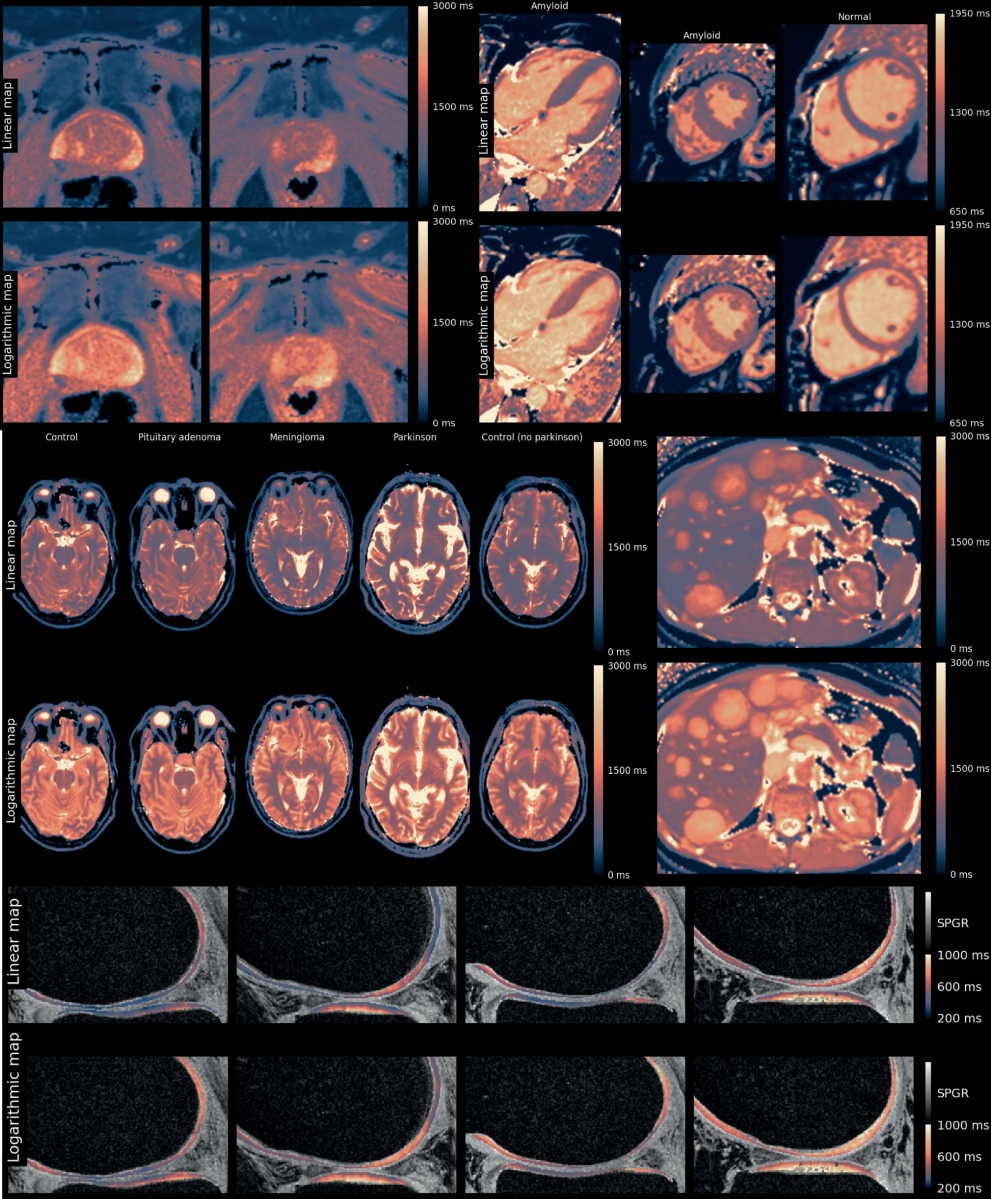
Collection of $T_{1}$ images as presented to the panel during Round 4. See Supporting Information for full‐sized images.

**FIGURE 4:**
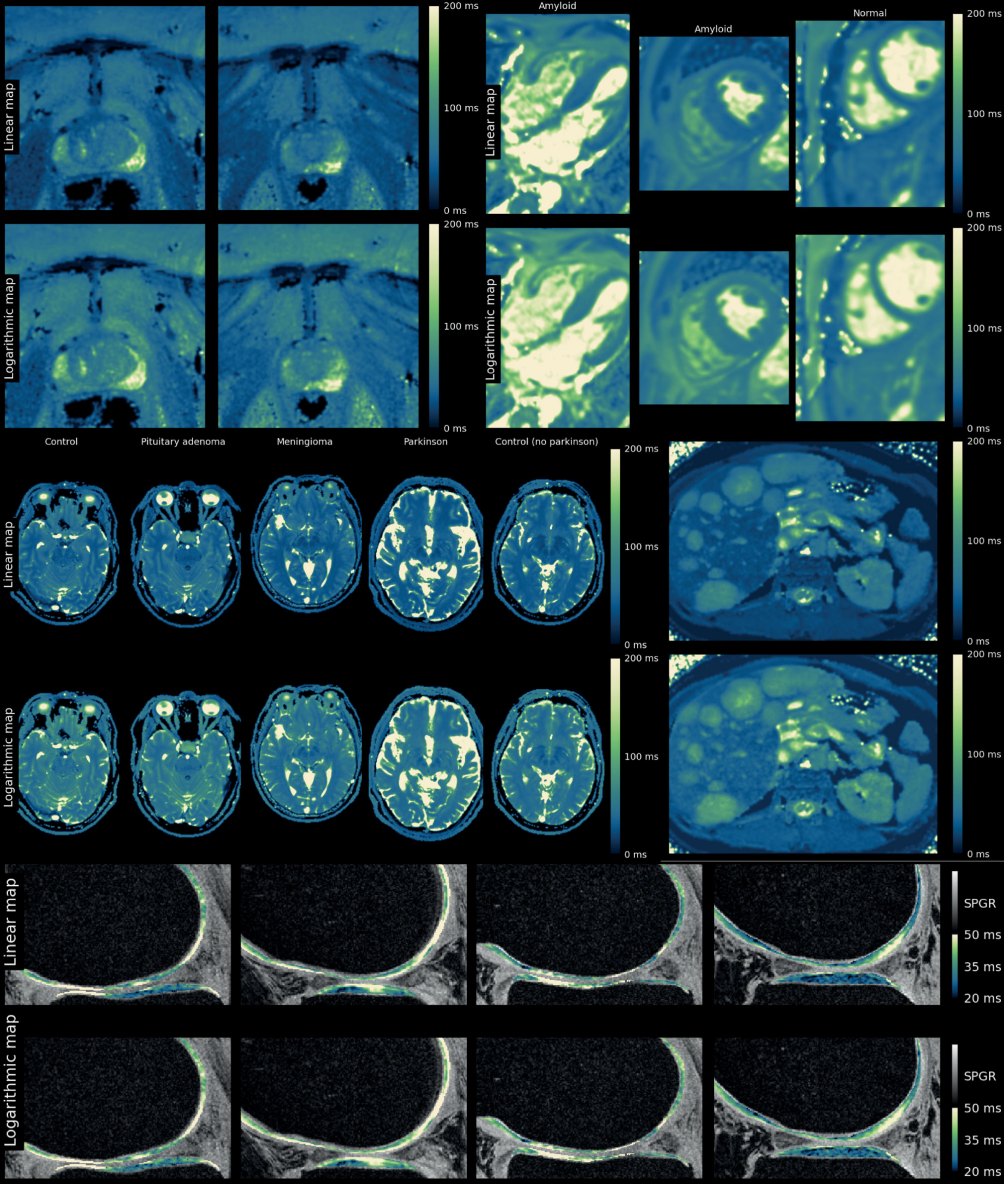
Collection of $T_{2}$ images as presented to the panel during Round 4. See Supporting Information for full‐sized images.

In Round 4, the Lipari and Navia maps were further refined based on the comments by the panel. Several example images were shown, which can best be seen in Supporting Information Data S3; Figures 3 and 4 show collections thereof.

### 2.3 Characteristics of the applied Delphi process

In most questions, the 9‐point Likert scale was summarized to a 3‐point Likert scale (“disagree” for scores 1, 2, and 3; “neutral” for scores 4, 5, and 6; and “agree” for scores 7, 8, and 9), where, according to Diamond et al.,^32^ a 75% consensus threshold was applied. This means that it was considered a consensus whenever the sum of scores 7, 8, 9 (or 1, 2, 3) reached 75% of all responses of that round. This a priori set threshold of 75% agreement was used, as it is a common choice in Delphi processes.^32^ The questions on “importance” were exceptions, such as “The proposed color‐maps should be as perceptually linear^16^ as possible.” For these questions, the 9‐point score ranged from “Not important” to “Critical,” and consensus was reached when the SD of the scores was below 2.0, allowing, for example, a consensus on “moderately important.”

The Delphi process in this study deviated from the conventional Delphi method, as the CRC occasionally modified questions from round to round. Regular CRC meetings took place during the process to discuss each round's outcomes and implement changes for subsequent rounds. These changes were motivated by several reasons. For example, during the process, the comments of some panelists indicated that a question was worded ambiguously; alternatively, remarks pointed to omissions in the set of questions. In other cases, the set of possibilities was evolving from broad to narrow. Finally, the suggested color‐maps were adapted from round to round, based on the comments by the panel.

### 2.4 Process behind the definition of the Lipari and Navia color‐maps

To create the two scientific color‐maps (Navia and Lipari; see Refs. 33 and 34), five evenly spread characteristic colors with variable lightness values were defined to resemble the overall look of the two color‐maps proposed in Griswold et al.^31^ To obtain a perceptually linear colormap based on the five anchor colors, the methodology of Crameri,^35^ as first outlined by Kovesi,^36^ was followed. These five colors were then complemented to a total of 256 individual colors following a smooth path within a perceptually uniform color space (here the L*a*b* color space according to the Commission Internationale de l' Eclairage^37^ [i.e., C.I.E.‐L*a*b* space, or “CIELAB” in brief]) passing these five chosen color values. In the following step, the perceptual differences between successive colors were calculated along the curve. From this, a cumulative sum of the perceptual differences along the color‐map was formed, which was then divided into 256 equally spaced values, which were then mapped back onto the original color‐space path via linear interpolation of the cumulative contrast curve. To form the final color‐map, this procedure was done repeatedly until the variation of local contrast values along the curve came below a predetermined threshold. For practical purposes, the L*a*b* values are transformed to sRGB (standardized Red, Green, Blue)^38^ values.

### 2.5 Perceptual linearity—relative to the relaxation values or to the logarithm thereof?

The aforementioned process^35^, ^36^ delivers a color‐map that is perceptually linear. If applied to the relaxation maps themselves, then the difference between $T_{2}$ = 20 ms and $T_{2}$ = 30 ms becomes as conspicuous as the difference between, say, 290 ms and 300 ms. The CRC questioned whether that is really the aim of the color‐map—or, whether according to the Weber‐Fechner's law,^39^ the difference between 20 ms and 30 ms should be as conspicuous as the difference between 200 ms and 300 ms (i.e., whether it is more meaningful to make the color‐map perceptually linear with respect to the *logarithm* of the relaxation value). This question was presented to the panel. This is the rationale behind the “lin‐log question” as entered in Delphi round 4. A possibility that was not considered at that time (see Section 4) was perceptual linearity with respect to $R_{1}$ or $R_{2}$.

However, “making the color map perceptually linear with respect to the logarithm of the value” does not involve taking the logarithm of the value maps. Rather, as outlined in Appendix 1, the color‐map is stretched such that it becomes perceptually linear with respect to the logarithm. The appendix also explains that, in order to avoid log(0), it is not really a pure logarithm. In the sequel, the color‐maps that are processed in this way are denoted as *logarithm‐processed Lipari* and *logarithm‐processed Navia*.^33^, ^34^

## 3 RESULTS

### 3.1 Panel composition

**FIGURE 5:**
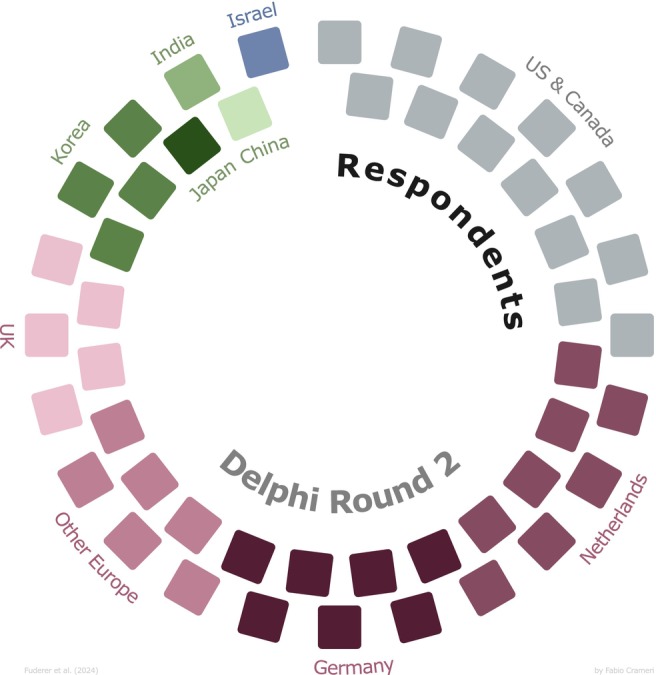
From Round 2, the number of respondents by country/region. Each block represents one person. Shades of purple reflect Europe; gray is North America; shades of green refer to Asia; and shades of blue refer to the Middle East. One responder preferred not to state his region, so only 47 responders are shown here.

The composition of the panel was assessed during Delphi Round 2 and had the following characteristics:

- Twenty‐two were medical professionals (45%) and 23 physicists (47%).
- Ninety‐six percent had a background in MRI (i.e., all but 2—1 of whom being an expert on color visualization).
- Years of experience ranged between 4 and 39, with an average of 19 years.
- For location (Figure 5), Europe (and particularly the Netherlands) was overrepresented (55%); China, India, and Japan were underrepresented (together, 6%), as well as Africa and Latin America (0%).
- One of the respondents (2%) had a type of color‐blindness.
- For gender, 24% were female (11), 76% were male (37), and 0% were other.

### 3.2 Delphi Round 1

The following items reached consensus during Round 1 (Table 1): The same color‐map should be used for all anatomies; a specific color needs to indicate invalidity; the choice of color scale and the software for the generation of color‐maps should be available for free; and perceptual linearity is important—also considering people with the most common type of color‐blindness (deuteranopia).

There was no consensus on the use of a single color scale for all relaxivity parameters, or whether two or more color scales would be needed for each relaxation property. Thus, the majority of items did not achieve consensus (Table 1), but the comments indicated that greater clarity was needed on range of colors and on perceptual linearity (Supplementary Material 4 in Data S4).

### 3.3 Delphi Round 2

Consensus was reached on the following points during Round 2: $R_{1}$ maps should have the same color scale (or inverse scale) as $T_{1}$ maps, and color‐maps should be as perceptually linear as possible—including *all* types of color‐impairment. On the range to be applied for color‐maps in the clinical applications, the responses came close to consensus; this did not apply to scientific work (Table 2). However, controversy remained around the display of $T_{1\rho }$ and whether $T_{2}$ and $T_{2}^{*}$ should be displayed similarly. There was also no consensus on whether the maps recommended by Griswold et al.^31^ should serve as the basis of these recommendations.

### 3.4 Delphi Round 3

Initial versions of the Lipari and Navia color‐maps were made available. This allowed presenting questions like “$T_{1}$ maps and $T_{2}$ maps should get the same color‐map” in a different way, showing examples. The questions on $T_{1\rho }$ were omitted because too many panel members were indifferent, so there was no prospect of a consensus.

The question on adaptable versus fixed minimum/maximum values for displaying the quantitative maps led to polarized opinions (see remarks in Supporting Information Data S4) with no consensus in sight. Thus, this question was dropped and references to ranges were omitted from the recommendations.

The panel's comments pointed to an omission regarding the color bar, and as a result, a new question on the necessity of a color bar was added.

Results are provided in Table 3. An interesting result thereof is the multiple‐choice question, in which 72% chose the “Lipari for $T_{1}$, Navia for $T_{2}$” option compared with 20% choosing the same mapping for both. The 72% fell short of the preset threshold of 75%, and a conventional Delphi process would necessitate repeating the question with the least popular choice (gray/gray) omitted. Yet, the CRC decided to treat the result as consensus, as the CRC deemed a future consensus very likely and they wanted to avoid increased complexity in Round 4.

**TABLE 3:** Results from Round 3.

Question	Score	Consensus?
(Number of respondents: 60)		
The [initial version of the] Lipari color‐map is suitable for T_1_ maps.	70% agreed	No
The [initial version of the] Navia color‐map is suitable for T_2_ maps.	53% agreed	No
Multiple choice for Lipari for T_1_, Navia for T_2_ (a); Lipari for T_1_ and Lipari for T_2_ (b); and gray for T_1_ and gray for T_2_ (c)	(a) 72% (b) 20% (c) 8%	No
T_2_ maps and $T_{2}^{*}$ maps should get the same color‐map.	80% agreed	Yes
Each quantitative relaxation image must be displayed in conjunction with a color bar with adequately readable numbers.	95% agreed	Yes
For clinical work, it is useful to have recommendations (per type of map and possibly per anatomy and field strength) on the range to be applied.	73% agreed	No

### 3.5 Delphi Round 4

**FIGURE 6:**
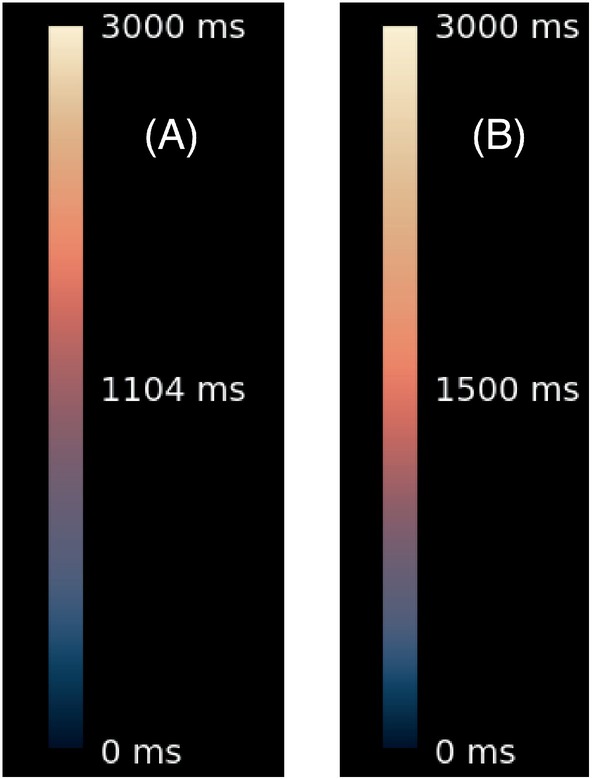
(A,B) The color bar and labeling as used in the questionnaire of Round 4 (A) and as used in the last, limited, questionnaire (B). The images were identical in both cases (e.g., 1500 ms being displayed as orange in both cases). (B) is the recommended choice.

The responses from Round 3 led to further adaptations. The Lipari and Navia maps were improved according to the comments received. As explained in Section 2, a question on logarithmic scale was entered here. Upon request, more examples were added, copies of which are provided in Supporting Information Data S3 (note that the color bars in these examples were not yet in the recommended form, as explained in Figure 6).

Two of the questions missed the threshold for consensus (Table 4): Suitability of Navia met 72% agreement (36 out of 50), and the question about linear versus logarithmic scaling (Lin‐Log) scored 70% (35 out of 50).

**TABLE 4:** Results from Round 4.

Question	Score	Consensus?
(50 respondents; 2 expressed their color‐blindness in a comment)		
The [improved version of the] Lipari color‐map is suitable for T_1_ maps.	75% agreed	Yes
The [improved version of the] Navia color‐map is suitable for T_2_ maps.	72% agreed	No
[New] The logarithmic maps are at least as suitable as linear maps.	70% agreed	No

When considering the Lin‐Log question, some of the respondents were confused by the wording of the question. Others were in favor of the “Log” images and the principle behind it but objected to the presentation of the color bar. (In the presented examples, the color bar in “Log” looked the same as in “Lin,” but the legend numbers were logarithmically spaced [Figure 6A].) As a result, the presentation of the color bar and its annotation were revised (Figure 6B) and the question was reformulated. Then, in another deviation to the strict Delphi process (see Section 4), the revision was not sent to the full panel, but only to those 8 respondents who were not unequivocally in favor of the logarithmic scaling and who identified themselves in the questionnaire. This resulted in 3 respondents in favor of a logarithmic scale and 5 in favor of a linear one. These 3 were added to the 35 respondents who approved the logarithmic scale in Round 4, bringing the total to 38 (i.e., 76%).

### 3.6 Summary of Delphi outcomes

Together, the following outcomes reached consensus:

- The color‐map should be independent of anatomy.
- A color‐map should contain a specific color (e.g., black), clearly distinguishable from all the other colors of the color‐map, to indicate invalidity (e.g., for regions of the image containing no tissue and therefore having no, or unknown, relaxation properties).
- $T_{2}$ maps and $T_{2}^{*}$ maps should have the same color‐map.
- $R_{1}$ maps should have the same color‐map as $T_{1}$ maps or the inverse thereof (an ambiguous wording, leaving two options open).
- Each quantitative relaxation image must be displayed in conjunction with a color bar with adequately readable numbers.
- The designed color‐maps, Lipari and Navia, have to be available for free and have to be as perceptually linear as possible—also when viewed by people with any kind of color‐blindness (i.e., perceptually linear when considering only the luminance component).
- Lipari is suitable for $T_{1}$; Navia is suitable for $T_{2}$ (Navia for $T_{2}$ was just below consensus threshold, but the CRC expected consensus in the next round).
- The color‐maps should be perceptually linear relative to the *logarithm* of the relaxation value.

## 4 DISCUSSION

### 4.1 Scope of this paper

Although this paper focuses on relaxation times, MRI can be used to generate many other quantitative imaging biomarkers. Elements of this Delphi study on relaxation‐related color‐maps might serve as an example that can be applied to other quantitative maps in future work.

### 4.2 Impact of color on contrast visibility

A benefit of using color is that it allows for more (color‐)contrast visibility than a grayscale image. This has been quantitatively analyzed using the ${\Delta E}_{\text{CIEDE}}$ metric,^16^ which expresses the perceptual contrast between neighboring values. This metric showed that the Lipari and Navia were superior to a grayscale map. Lipari and Navia scored an average ${\Delta E}_{\text{CIEDE}}$ of about 0.44 against 0.29 for a grayscale map. Thus, according to theory, Lipari and Navia should both show better contrast visibility than grayscale throughout the whole range. Experimental confirmation thereof would require an extensive perception experiment, which was not part of this study. The measurement point we do have, however, is that 43 participants chose the Lipari/Navia option compared with 5 for the gray/gray option in Round 3, which was before further finetuning of the Navia color‐map.

Although a color‐map like Jet may still have a higher average ${\Delta E}_{\text{CIEDE}}$ score, this comes at the cost of locally over‐enhancing or suppressing contrasts and at the cost of being non‐monotonic in luminance (the brightest color, yellow, being halfway the range), therefore not making it easily interpretable by color‐impaired viewers.

### 4.3 Displaying the units

Some details of the recommendation were retrospectively added by the CRC. In Round 3, the following statement reached clear consensus: “Each quantitative relaxation image must be displayed in conjunction with a color‐bar with adequately readable numbers.” In retrospect, the CRC realized that the intended statement should have been, “Each quantitative relaxation image must be displayed in conjunction with a color‐bar with adequately readable numbers *and units*.” Although the addition “*and units*” is—strictly speaking—not a Delphi outcome, the CRC has sufficient confidence that such addition would not have altered the consensus. This addition is reflected in the summary and the conclusions.

### 4.4 Shortcuts used after Round 4

After Round 4, a small‐scale questionnaire on the Lin‐Log question was submitted to 8 participants, because the declining number of responders with each round indicated respondent fatigue and did not justify a subsequent “full” repeat round. Therefore, the extra round was limited to all non‐anonymous participants who had not agreed to logarithmic maps in Round 4 and who had indicated they were unclear about the question being asked. In this extra round on clarifying the question, 3 of the 8 responded positively to the logarithmic maps, taking the total score of those preferring logarithmic maps to 76%.

For the same reason of responder fatigue, no further iterations on the Navia color‐map were done after Round 4. In Round 4, agreement to Navia scored 36 of 50 (or 72%); only 2 panel members disagreed (the remaining 12 were indifferent). One of these 2 was a respondent who systematically indicated—in contrast to all the others—a lack of interest in display of a quantitative map but only an interest in *interpretation* of the quantitative map (with color‐labeled “normal” or “abnormal”). The case for Navia was further strengthened by 2 color‐blind panel members in Round 4, who were in favor of the Navia color‐map. Given this situation, Navia was accepted as a consensus even though it formally achieved a 72% agreement.

Finally, the last question (“useful to have recommendations […] on the range”) was also very close to consensus (73%), so it would very likely result in full consensus in the next round. However, during the discussion, it was realized that recommendations about ranges, to be useful, needed to be set on an organ‐by‐organ basis, with very clear directions from specialists in every single application of quantitative relaxometry. As the CRC was composed of a subgroup of such specialists only, it was decided not to pursue this recommendation for the time being. Although range recommendations are relevant, they remain future work.

### 4.5 Community‐submitted discussion points

The following discussion points were entered by attendants to the qMR‐SG meeting at the ISMRM 2023 or those submitted as part of the endorsement process.

- One panel member systematically rejected the proposed color‐maps; in this person's view, color should be used to indicate normal versus abnormal tissue. This idea is interpreted as a suggestion on a color‐map for an interpretation or segmentation of the tissue (which may be based on quantitative maps) but not as a color‐map for the quantitative ($T_{1}$ or $T_{2}$) maps themselves.
- Ideally, we would like to have perceptual linearity with respect to clinically relevant histological parameters, rather than to $T_{1}$ or $T_{2}$ directly. As an example, $R_{1}$ (or $R_{2}$) may relate to the physical quantity of contrast uptake; regarding this example, it would be most logical to make a color‐map perceptually linear with respect to $1/T_{1}$, rather than $\log\left(T_{1}\right)$. For many other examination types, there may be other nonlinear relationships between relaxation parameters and underlying physiology. It is impossible to encompass all possible nonlinearities simultaneously. Specifically on the contrast uptake, the argument was only recognized after closure of the Delphi process. Furthermore, the difference between $R_{1}$ and $\log\left(T_{1}\right)$ curves would be slight, as both for $1/T_{1}$ and for $\log\left(T_{1}\right)$, a deviation from the theoretical curve is required for small $T_{1}$ values (see Appendix 1). Further modifications to the current Delphi outcome are considered as future work.
- Perceptual linearity is, in principle, guaranteed in the sRGB color space (i.e., the color‐maps are perceptually linear for a default display device as defined by the CIE,^38^ for the “average” display system). In practice, no two display devices are truly identical, particularly considering (mis‐)adjustments and (for liquid‐crystal displays) viewing‐angle dependencies.
- The “invalid” color has been chosen as black. This has been hinted at in the Delphi process statement: “A color‐map should contain a specific color (e.g., black), clearly distinguishable from all the other colors of the color‐map, to indicate invalidity.” However, the “e.g.” still leaves some room for choices. Yet, any monochromatic choice, other than black or white, could be seen by color‐impaired people as one of the “valid” colors. A pattern (e.g., a checkerboard) would indicate invalidity more clearly, but it would complicate the viewing pipeline, and it would not conform to the statement presented in the Delphi process. Between black and white, the CRC chooses black, because it causes less glare. Care has been taken that the first valid color is perceptually at least 10% distant from black.
- In the demographics question, only 1 participant declared to be color‐blind (2%). This is below the average prevalence of the condition. In retrospect, for this study, it would have been beneficial to have more color‐blind people participating. Yet, it is difficult to actively select panel members on this condition—as opposed to, for example, the academic degree; color‐blindness is rarely shared with a wide audience.

### 4.6 Future work

While consensus was achieved on many points, the divergence of opinions among experts left a number of issues unresolved. In particular, the question on “The range of an applied color‐map should [not] be fixed” did not reach consensus, with clear argumentation by both proponents and opponents on each side. Its alternative, “It is useful to have recommendations (…) on the range” came close to consensus; yet, as discussed, it was decided not to pursue this recommendation for the time being. While range recommendations are relevant, they remain future work.

Although the benefit of Lipari and Navia has been established *in theory* via a superior average value of ${\Delta E}_{\text{CIEDE}}$ compared with gray‐value maps, this superiority has not been experimentally validated, which would be a valuable addition to this work.

**FIGURE 7:**
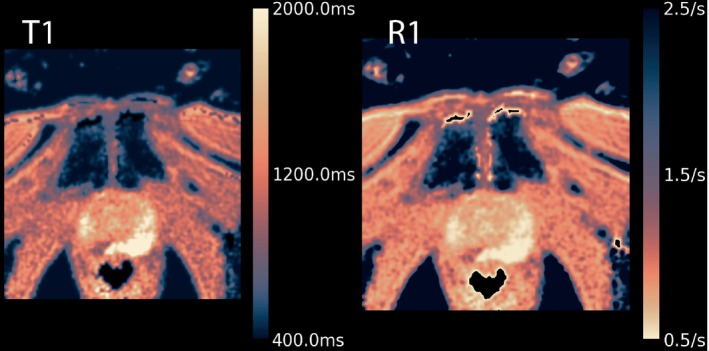
Example of a prostate T_1_ map (*left*) and the corresponding R_1_ map (*right*) displayed using the logarithm‐processed inverted Lipari color‐map. Given the logarithm processing, with the inverted map, the R_1_ looks almost identical to the T_1_ map. Note that the logarithm is not applied to the maps themselves but by processing the color‐map. This is apparent by the difference between the color bars: The color corresponding to the $T_{1}$ value that is linearly halfway (400 ms and 2000 ms) is much brighter than the color corresponding to the $R_{1}$ value that is linearly halfway (0.5/s and 2.5/s).

Another question that reached consensus but remained ambiguous was worded as “$R_{1}$ maps should get the same color‐map as $T_{1}$ maps, or the inverse thereof.” The remaining ambiguity was never resolved. Consequently, it was recommended to use Lipari for $R_{1}$ and Navia for $R_{2}$, but it is explicitly left open on whether the maps should be reversed when applying these to $R_{1}$ or $R_{2}$. Both choices have proponents: On the one hand, it is intuitive to systematically associate high values with bright image regions; on the other hand, a reversed map would mean that one gets almost the same image when applying a logarithmic display of a $T_{1}$ map compared with the inverted logarithmic display of the associated $R_{1}$ map. The latter is shown in Figure 7.

The polling on $T_{1\rho }$ was dropped from the Delphi questionnaire after Round 2, as the responses indicated indifference among the panel members. This indifference may be partly related to the fact that the term $T_{1\rho }$ does not describe a scalar value but actually represents a (lock field–dependent) continuum of values for any single tissue.^40^ Thus, to reach a consensus on $T_{1\rho }$, a detailed discussion in which the context of use and a rigorous physical definition are provided may be needed; this is beyond the scope of the present work.

## 5 CONCLUSION

As outlined in the resulting recommendations (Table 5), the CRC and the endorsers are confident in recommending the logarithm‐processed Lipari color‐map for $T_{1}$ maps and the logarithm‐processed Navia color‐map for $T_{2}$‐like maps. Combined with the recommendation that each quantitative relaxation image be displayed in conjunction with a color bar, this will lead to more uniformity, more comparability across results, and easier recognizability of the map type.

**FIGURE 8:**
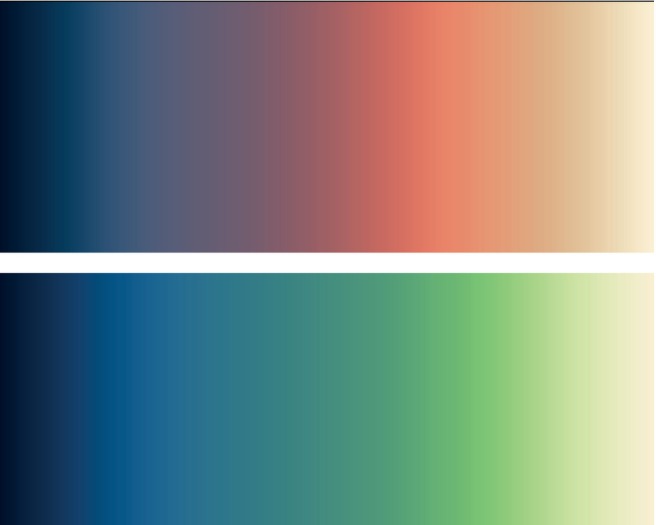
The recommended (unprocessed) color‐maps: Lipari (*top*) and Navia (*bottom*).

See Figure 8 for the unprocessed maps.

**TABLE 5:** Resulting recommendations.

The logarithm‐processed Lipari color‐map should be used for $T_{1}$ maps, and the logarithm‐processed Navia color‐map should be used for $T_{2}$ and $T_{2}^{*}$. The color‐maps and the logarithm processing are jointly available in https://doi.org/10.5281/zenodo.8268884. The same color‐maps are to be applied on $R_{1}$, $R_{2}$, and $R_{2}^{*}$, respectively. This recommendation holds for all anatomies. The value of 0 is to be used to indicate that the calculated relaxation value is unknown (or “invalid”) at that specific pixel, and it always should map to black. In addition, each quantitative relaxation image should be displayed in conjunction with a color bar with adequately readable numbers and units. These recommendations, which apply both to commercial display systems as well as to scientific publications, only achieve the aimed benefits with wide adoption. From this follows a plea on industry to adopt the recommendations, on the scientific community to use these recommendations when internally communicating MR relaxation maps, and a fortiori when publishing these. Similarly, colleagues are encouraged to promote the use of these recommendations, such as when peer‐reviewing.

## CONFLICT OF INTEREST

Ruud de Boer was, at the time of writing, an employee of Philips. Miha Fuderer's project has been financed by NWO grant number 17986, which has partly been funded by the company Philips. Xavier Golay is a founder, shareholder, and employee of Gold Standard Phantoms. Xavier Golay is a consultant at Bioxydyn. Vikas Gulani receives research support from Siemens Healthineers and has intellectual property licensed by Siemens Healthineers. Dan Ma has patents licensed by Siemens. Carolin Pirkl is employed by GE HealthCare. Barbara Wichtmann has given scientific presentations for Philips, Lilly Deutschland, and for Bender group/b.e.imaging GmbH on unrelated topics; for these presentations, monetary compensation was received.

## Supporting information


**Data S1.** The variety of color‐maps used in current literature on proton density maps.

**Data S2.** The variety of color‐maps used in current literature on relaxation.

**Data S3.** The images as presented in the questionnaire of Round 4 of the Delphi process.

**Data S4.** Collection of received comments.

**Data S5.** The list of endorsers.

## Data Availability

The color‐maps and the logarithm processing are jointly available at https://doi.org/10.5281/zenodo.8268884.

## APPENDIX 1

### Details of the “logarithm” lookup

The recommendation is to map these color ranges to the *logarithm* of $T_{1}$ or $T_{2}$ values, rather than to the $T_{1}$ or $T_{2}$ values themselves.

However, this does not mean that an explicit logarithm has to be applied; rather, the proposal is to process the Lipari or Navia maps in such a way that their processed versions become perceptually linear to the logarithm of the input rather than being perceptually linear to the input (see Figure 6B). As there are no recommendations on the ranges of $T_{1}$ or $T_{2}$, the lower level of the scale may be zero (which makes the logarithm ill‐defined) or even negative. A negative lower level makes no sense if the display device is well‐calibrated and the color‐map is perceptually linear; however, if this ideal situation is not met, some users may be tempted to set the lower level to a negative value—and most user interfaces do allow it.

For that purpose, a parametrized function is defined as having a linear segment and a logarithmic segment. In the following, it is assumed that:

- A pixel with a value of zero represents “invalid/unknown” (i.e., the quantitative value is unknown at that pixel).
- The values of L and U represent the lower and upper end of the range of values to be displayed. For U, it is assumed that it is strictly positive; for L, no such assumption holds.
- The value of ϵ represents the smallest value that represents a meaningful relaxation value. The relaxation value that this may refer to, may depend on the rescale slope (in DICOM terms); for example, the stored pixel values may range from 0 to 255 but actually represent $T_{1}$ values ranging from 0 to 2000 ms. In that example, the rescale slope is 7.843, and ϵ (which corresponds to a stored value of 1) equals 7.843.
- The output f(x) ranges from 0 to 1, which can be scaled arbitrarily to the length of the color‐map (e.g., multiplying by 511.99 for a 512‐entry table). Here, f(x)=0 defines the “invalid” color (i.e., black). Here, $\epsilon_{f}$ refers to the value of f that maps to the smallest nonzero color entry (in this example, to 1/511).
- The auxiliary variable a, which separates the linear part from a logarithmic part, is declared as a=U·exp(−1).
- The auxiliary variable b, which equals f(x) for x=max(a,L), is calculated as $b=\left\{\begin{array}{ll} \frac{a-L}{2a-L}+\epsilon_{f} & a\geq L\\ \epsilon_{f} & a\leq L \end{array}\right.$ here, with
  $$f\left(x\right)=\left\{\begin{array}{ll} 1 & U\leq x\\ \frac{\left[\ln(x)-\ln\left(\max\left(a,L\right)\right)\right]}{\left[\ln(U)-\ln\left(\max\left(a,L\right)\right)\right]}\left(1-b\right)+b & \max\left(a,L\right)\leq x\leq U\\ \left[\left(x-L\right)/\left(a-L\right)\right]\left(b-\epsilon_{f}\right)+\epsilon_{f} & \max\left(0,L\right)<x\leq a\;\text{if}\;L<a\\ \epsilon_{f} & 0<x\leq L\;\text{if}\;0<L\\ 0 & x=0 \end{array}\right.$$

The Lipari and Navia maps themselves are publicly available.^41^ The tooling to apply the logarithm processing, and to apply the results to images, is available in *MATLAB* ^34^ or *Julia*.^33^
