# Supplementary material for: Color‐map recommendation for MR relaxometry maps
Source: Magn Reson Med. 2024 Oct 16;93(2):490–506. doi: 10.1002/mrm.30290 (PMC11604837; doi:10.1002/mrm.30290)
Supplement: Supplementary file 1 — Data S1. The variety of color‐maps used in current literature on proton density maps. [file MRM-93-490-s001.docx]

# Supplementary 1

The current variety of color-maps used in literature was assessed for proton-density images (PD).

Method: Using scholar.google.com, the search term “MRI quantitative proton density mapping” was applied, combined with the restriction on publication-year>=2022. This resulted in 17,000 hits, which were sorted according to ‘relevance’ (in this case, ‘relevance’ refers to mutual proximity of the search terms, not necessarily scientific relevance).

Then these were filtered on those that the first author (MF) had access to, on *not* featuring proton density fat fraction (PDFF) and actually displaying at least one proton-density map as an image. Further, duplications on the first author were omitted. From the filtered set, the first 10 were considered.

Results: The scholar.google survey on proton-density resulted in the usage as reported in Table S2. The predominant color map for PD seems to be Grey (7 out of 10), followed by the use of Jet.

| **First author** | **Color map for PD** | **Remarks** |
| --- | --- | --- |
| Jara^1^ | Grey | Uses grey for all of PD, T1, T2, R1, R2 |
| Sila Dokumaci^2^ | Grey | Uses grey for both PD and T1 |
| Saito^3^ | Grey/other | Uses grey in figure 4 and a blue-ish map in figure 5 |
| Zheng^4^ | Jet | Uses jet for all of T1, T2, PD |
| Buch^5^ | Grey |  |
| Gao^6^ | Jet | Uses jet for all of T1, T2, PD; it is a bit odd that the PD image (fig 2b) is expressed in ms. |
| McDowell^7^ | Grey | Uses grey for all of T1, T2, PD |
| Seif^8^ | Grey | Uses grey for all of T1, T2, PD |
| Cao^9^ | Grey | (T1, T2 and ADC in color) |
| Luo^10^ | Jet | Uses jet for all of T1, T2, PD |
| Table S1: Variety of color-maps used for proton-density in recent literature | | |

Conclusion: In scientific literature, PD maps are predominantly displayed using the greyscale map.

References

1. Jara H, Sakai O, Farrher E, et al. Primary Multiparametric Quantitative Brain MRI: State-of-the-Art Relaxometric and Proton Density Mapping Techniques. *Radiology*. 2022;305(1):5-18. doi:10.1148/radiol.211519

2. Sila Dokumaci A, Vecchiato K, Tomi-Tricot R, et al. Quantitative T1 and Effective Proton Density (PD*) mapping in children and adults at 7T from an MP2RAGE sequence optimised for uniform T1-weighted (UNI) and FLuid And White matter Suppression (FLAWS) contrasts. *medRxiv*. Published online January 1, 2024. Accessed July 13, 2024. http://medrxiv.org/content/early/2024/07/01/2024.06.28.24307535.abstract

3. Saito M. MRI‐based quantification of carbon and oxygen concentrations in human soft tissues for range verification in proton therapy. *Med Phys*. 2023;50(9):5671-5681. doi:10.1002/mp.16353

4. Zheng Z, Liu Y, Yin H, et al. Evaluating T1, T2 Relaxation, and Proton Density in Normal Brain Using Synthetic MRI with Fast Imaging Protocol. *Magnetic Resonance in Medical Sciences*. Published online 2023:tn.2022-0161. doi:10.2463/mrms.tn.2022-0161

5. Buch S, Subramanian K, Chen T, et al. Characterization of white matter lesions in multiple sclerosis using proton density and T1-relaxation measures. *Magn Reson Imaging*. 2024;106:110-118. doi:10.1016/j.mri.2023.12.004

6. Gao W, Yang Q, Li X, et al. Synthetic MRI with quantitative mappings for identifying receptor status, proliferation rate, and molecular subtypes of breast cancer. *Eur J Radiol*. 2022;148:110168. doi:10.1016/j.ejrad.2022.110168

7. McDowell AR, Petrova N, Carassiti D, et al. High‐resolution quantitative MRI of multiple sclerosis spinal cord lesions. *Magn Reson Med*. 2022;87(6):2914-2921. doi:10.1002/mrm.29152

8. Seif M, Leutritz T, Schading S, et al. Reliability of multi-parameter mapping (MPM) in the cervical cord: A multi-center multi-vendor quantitative MRI study. *Neuroimage*. 2022;264:119751. doi:10.1016/j.neuroimage.2022.119751

9. Cao X, Liao C, Zhou Z, et al. DTI‐MR fingerprinting for rapid high‐resolution whole‐brain T1, T2 , proton density, ADC, and fractional anisotropy mapping. *Magn Reson Med*. 2024;91(3):987-1001. doi:10.1002/mrm.29916

10. Luo XW, Li QX, Shen LS, et al. Quantitative association of cerebral blood flow, relaxation times and proton density in young and middle-aged primary insomnia patients: A prospective study using three-dimensional arterial spin labeling and synthetic magnetic resonance imaging. *Front Neurosci*. 2023;17. doi:10.3389/fnins.2023.1099911
