# Supplementary material for: Color‐map recommendation for MR relaxometry maps
Source: Magn Reson Med. 2024 Oct 16;93(2):490–506. doi: 10.1002/mrm.30290 (PMC11604837; doi:10.1002/mrm.30290)
Supplement: Supplementary file 2 — Data S2. The variety of color‐maps used in current literature on relaxation. [file MRM-93-490-s003.docx]

# Supplementary 2

The current variety of color-maps used in literature was assessed.

Figure S2: Color-maps encountered in recent MR literature.


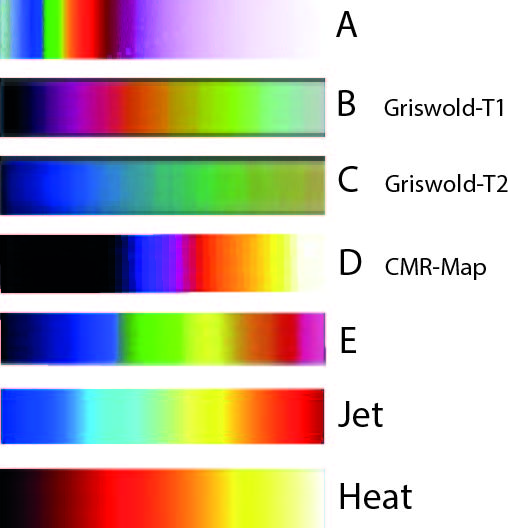


Method: Using scholar.google.com, the search term “quantitative T1 T2” was applied, combined with the restriction on publication-year>=2022. This resulted in 19400 hits, which were sorted according to ‘relevance’ (in this case, ‘relevance’ refers to mutual proximity of the search terms, not necessarily scientific relevance).

Then these were filtered on those that the first author (MF) had access to, and on publications showing any of a quantitative $T_{1}$ or a quantitative $T_{2}$ map as an image. Further, duplications on the first author were omitted.

Note that we had to guess the name of the color mapping from the color bar (if any). For some of these, this is not straightforward. These are shown in Figure S1; in some instances, we added the *likely* name of the color-map.

Results: The scholar.google survey resulted in the usage as reported in Table S1.

| **First author** | **Image type** | **Color-map used** |
| --- | --- | --- |
| Serai^1^ | T1 | Figure S1 A |
| Qiao^2^ | T1, T2, T2* | Jet |
| Boushina^3^ | T1, T2, T2* | Jet |
| Velasco^4^ | T1 | Figure S1 B  (Griswold-T1?) |
|  | T2, T2*, T1rho | Figure S1 C  (Griswold-T2?) |
| Gao^5^ | T1,T2 | Jet |
| Konar^6^ | T1, T2 | Jet |
| Hänninen^7^ | T2 | Jet |
| Qian^8^ | T1, T2 | Heat |
| Peng^9^ | T1 | Figure S1 D |
| Sen Ma^10^ | T1 | Jet |
|  | T2, T1rho | Figure S1 D |
| Yamada^11^ | T2 | Figure S1 E |
| Mahalingam^12^ | T1 | Figure S1 A |
| Sharafi^13^ | T1, T2, T1rho | Jet |
| Johnson^14^ | T1rho, T2 | Jet |
| Iwata^15^ | T2 | (no bar) (Jet??) |
| Table S2: Variety of color-maps used in recent literature | | |

Discussion: Meanwhile, in other publications, e.g., by a random pick from abstracts at the ISMRM 2022 and 2023, the usage of Gist_stern, Viridis, Spectral and Inferno was observed, adding four more to the above.

Note that “Jet” seems to be popular in recent publications. Yet, by (accidentally) omitting the constraint “year>=2022” from our search, different color-maps were observed in the six ‘most relevant’ hits: Figure S1 map D, Figure S1 map F, and four greyscale-maps. This suggests a gradual trend from displaying quantitative relaxation maps in greyscale (despite the very early use of color in 1978) towards a usage of a variety of color-maps.

From this, we conclude that there is a wide variety of applied color-maps for relaxation-maps in recent MR literature.

References

1. Serai SD. Basics of magnetic resonance imaging and quantitative parameters T1, T2, T2*, T1rho and diffusion-weighted imaging. doi:10.1007/s00247-021-05042-7/Published

2. Qiao H, Yang Q, Huo R, et al. Reliability and Value of 3D Sequential QUantitative T 1-T 2-T 2 * MAppings (SQUMA) MR Multi-Parametric Imaging in Characterizing Carotid Artery Atherosclerosis. Published online 2022. doi:10.1002/jmri.28445

3. Bouhsina N, Decante C, Hardel JB, et al. Comparison of MRI T1, T2, and T2* mapping with histology for assessment of intervertebral disc degeneration in an ovine model. *Scientific Reports |*. 123AD;12:5398. doi:10.1038/s41598-022-09348-w

4. Velasco C, Cruz G, Jaubert O, et al. T E C H N I C A L N O T E , T 1ρ , and fat fraction characterization with MR fingerprinting Magnetic Resonance in Medicine published by Wiley Periodicals LLC on behalf of International Society for Magnetic Resonance in Medicine. mapping, liver MRI, magnetic resonance fingerprinting, multiparametric, T 1⍴ mapping, T1 mapping, T2 mapping. *Magn Reson Med*. 1980;87:1980-1991. doi:10.1002/mrm.29089

5. Gao W, Yang Q, Li X, et al. Synthetic MRI with quantitative mappings for identifying receptor status, proliferation rate, and molecular subtypes of breast cancer. *Eur J Radiol*. 2022;148:110168. doi:10.1016/J.EJRAD.2022.110168

6. Shridhar Konar A, Deelip Shah A, Paudyal R, et al. Quantitative Synthetic Magnetic Resonance Imaging for Brain Metastases: A Feasibility Study. Published online 2022. doi:10.3390/cancers14112651

7. Hänninen NE, Liimatainen T, Hanni M, et al. Relaxation anisotropy of quantitative MRI parameters in biological tissues. *Scientific Reports |*. 123AD;12:12155. doi:10.1038/s41598-022-15773-8

8. Qian E, Poojar P, Thomas Vaughan Jr J, Jin Z, Geethanath S. Tailored magnetic resonance fingerprinting for simultaneous non-synthetic and quantitative imaging: A repeatability study. Published online 2022. doi:10.1002/mp.15465

9. Peng F, Xu H, Song Y, et al. Utilization of T1-Mapping for the pelvic and thigh muscles in Duchenne Muscular Dystrophy: a quantitative biomarker for disease involvement and correlation with clinical assessments. Published online 2021. doi:10.1186/s12891-022-05640-y

10. Ma S, Wang N, Xie Y, et al. Motion-robust quantitative multiparametric brain MRI with motion-resolved MR multitasking for Magnetic Resonance in Medicine. Published online 2021. doi:10.1002/mrm.28959

11. Yamada H, Tanikawa M, Sakata T, Aihara N, Mase M. Usefulness of T2 Relaxation Time for Quantitative Prediction of Meningioma Consistency. *World Neurosurg*. 2022;157:e484-e491. doi:10.1016/J.WNEU.2021.10.135

12. Mahalingam N, Trout AT, Gandhi DB, et al. Associations between MRI T1 mapping, liver stiffness, quantitative MRCP, and laboratory biomarkers in children and young adults with autoimmune liver disease. 2022;47:672-683. doi:10.1007/s00261-021-03378-0

13. Sharafi A, Zibetti MVW, Chang G, Cloos M, Ravinder |, Regatte R. 3D magnetic resonance fingerprinting for rapid simultaneous T1, T2, and T1ρ volumetric mapping of human articular cartilage at 3 T. Published online 2022. doi:10.1002/nbm.4800

14. Johnson CP, Tóth F, Carlson CS, et al. T1ρ and T2 mapping detect acute ischemic injury in a piglet model of Legg-Calvé-Perthes disease. *J Orthop Res*. 2022;40:484-494. doi:10.1002/jor.25044

15. Iwata S, Yawara Eguchi ·, Takaoka H, et al. MRI T2-mapping of lumbar facet joints is effective for quantitative evaluation of lumbar instability in patients with degenerative lumbar disorders. *European Spine Journal*. 2022;31:1479-1486. doi:10.1007/s00586-022-07119-9
