# Supplementary material for: Color‐map recommendation for MR relaxometry maps
Source: Magn Reson Med. 2024 Oct 16;93(2):490–506. doi: 10.1002/mrm.30290 (PMC11604837; doi:10.1002/mrm.30290)

# Supplementary 3


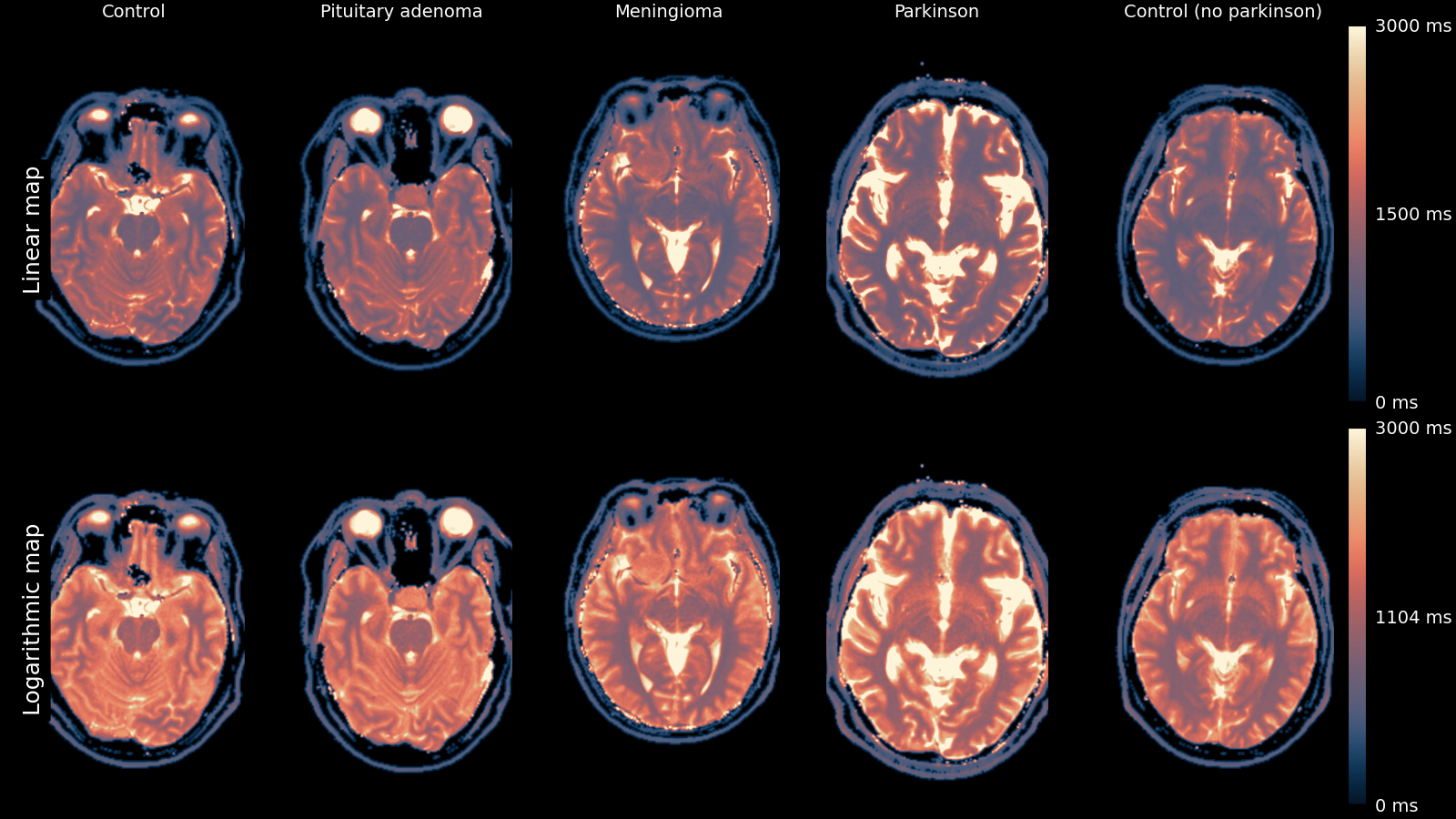
The images as presented in the questionnaire of round 4 of the Delphi process.


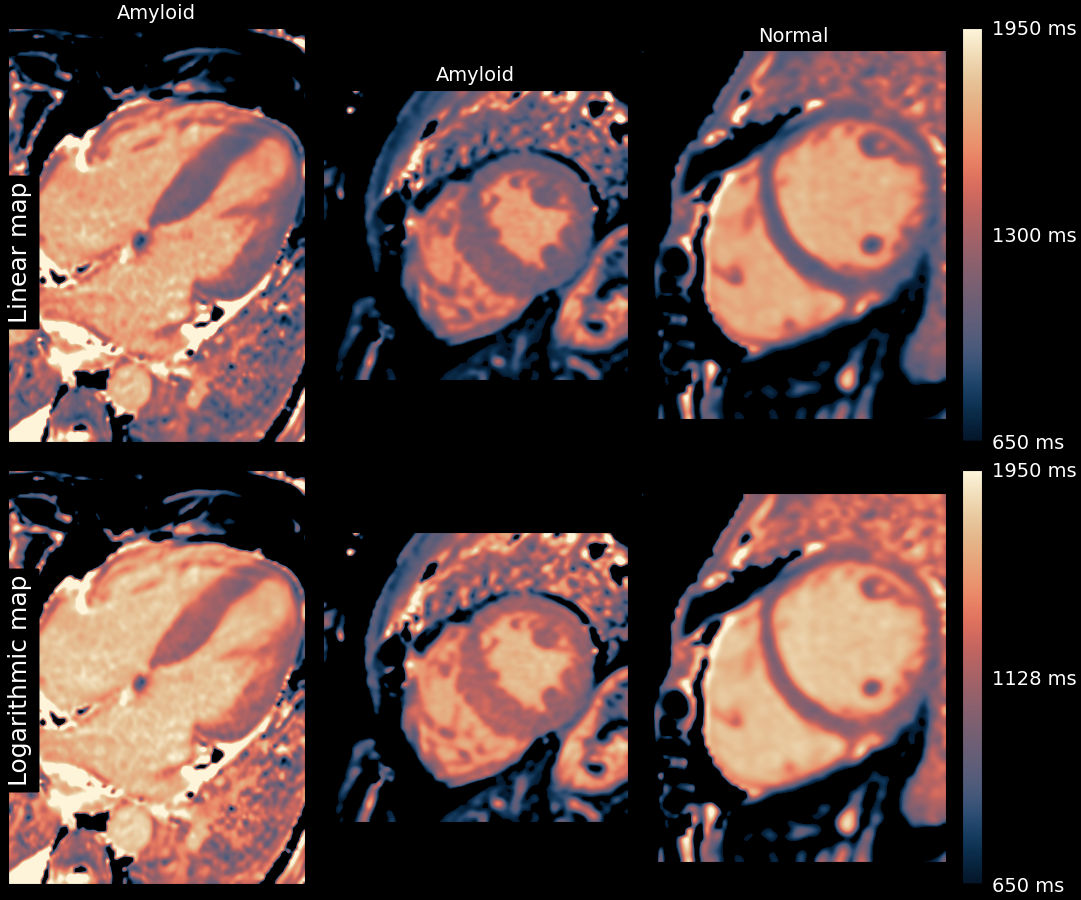


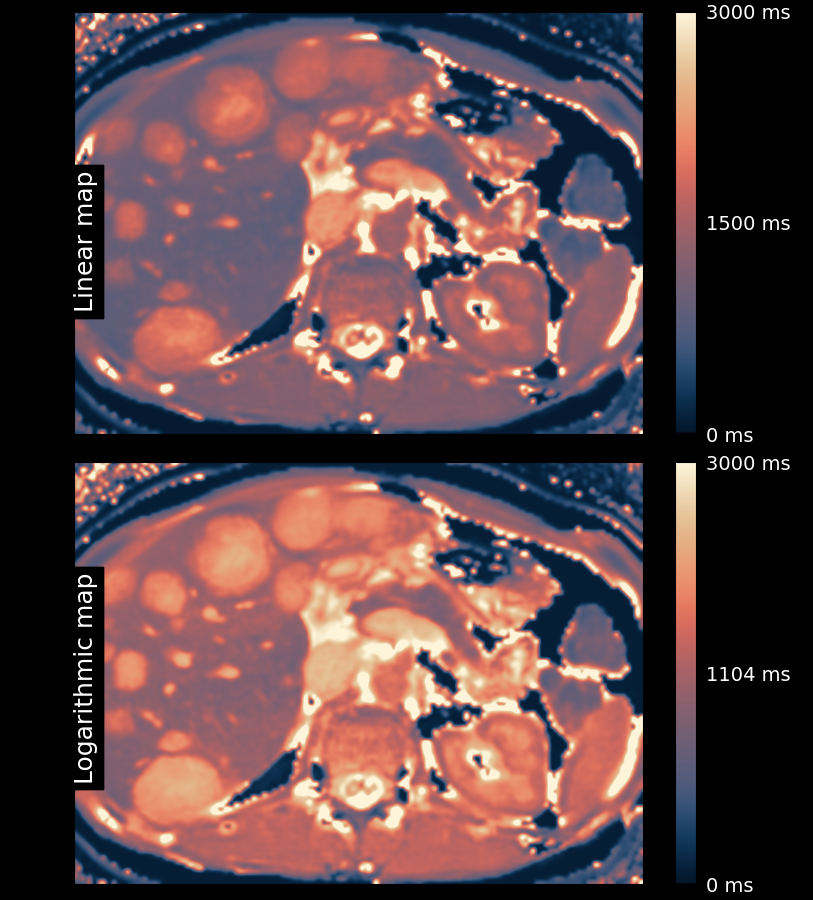


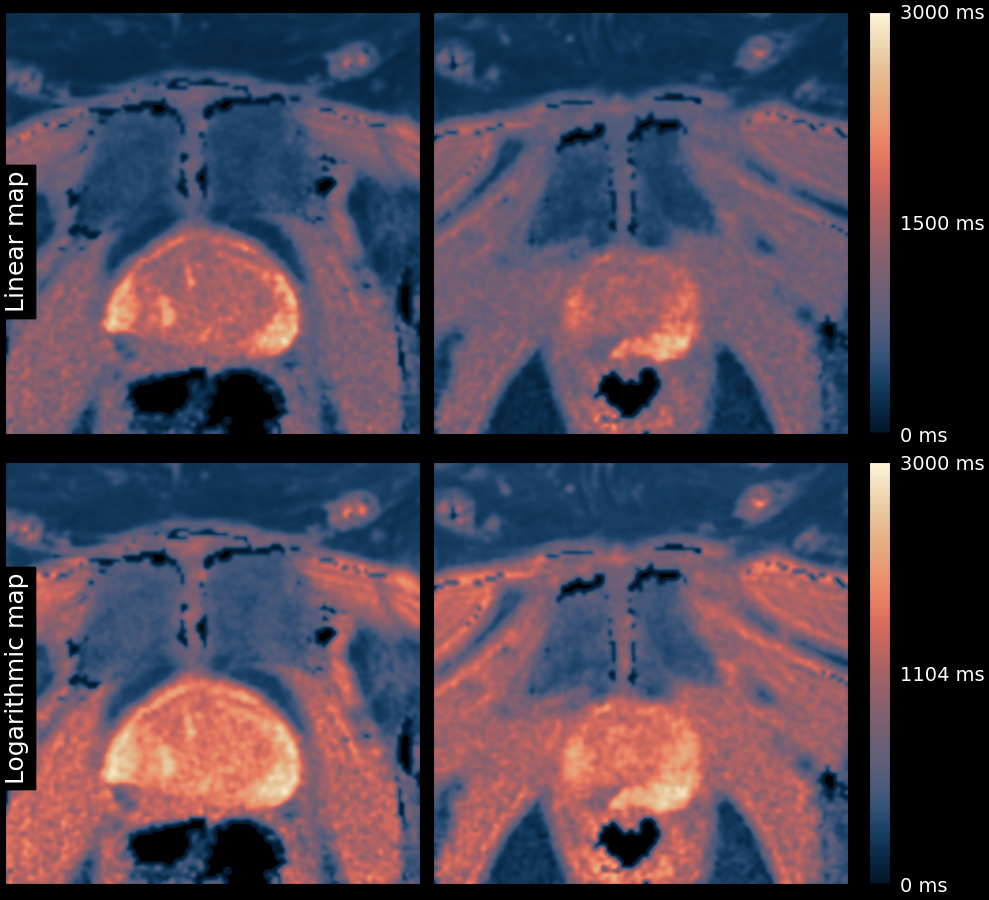


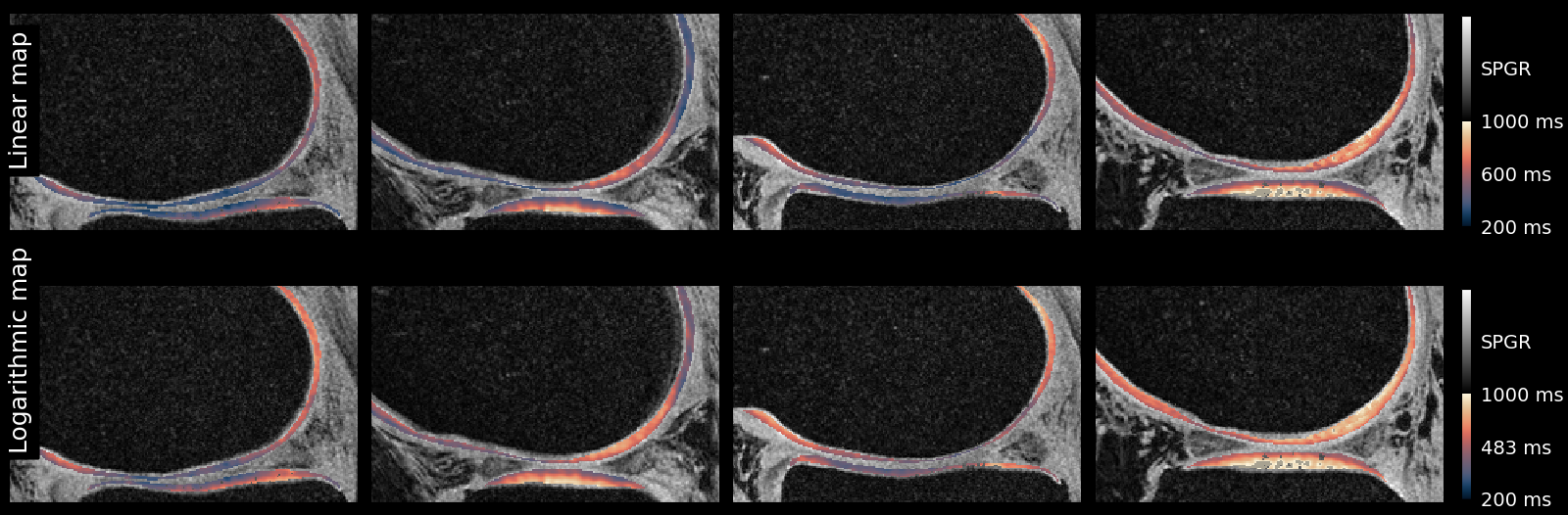


### Data courtesy Dirk Poot and Edwin Oei, Erasmus MC, QUICK Study.


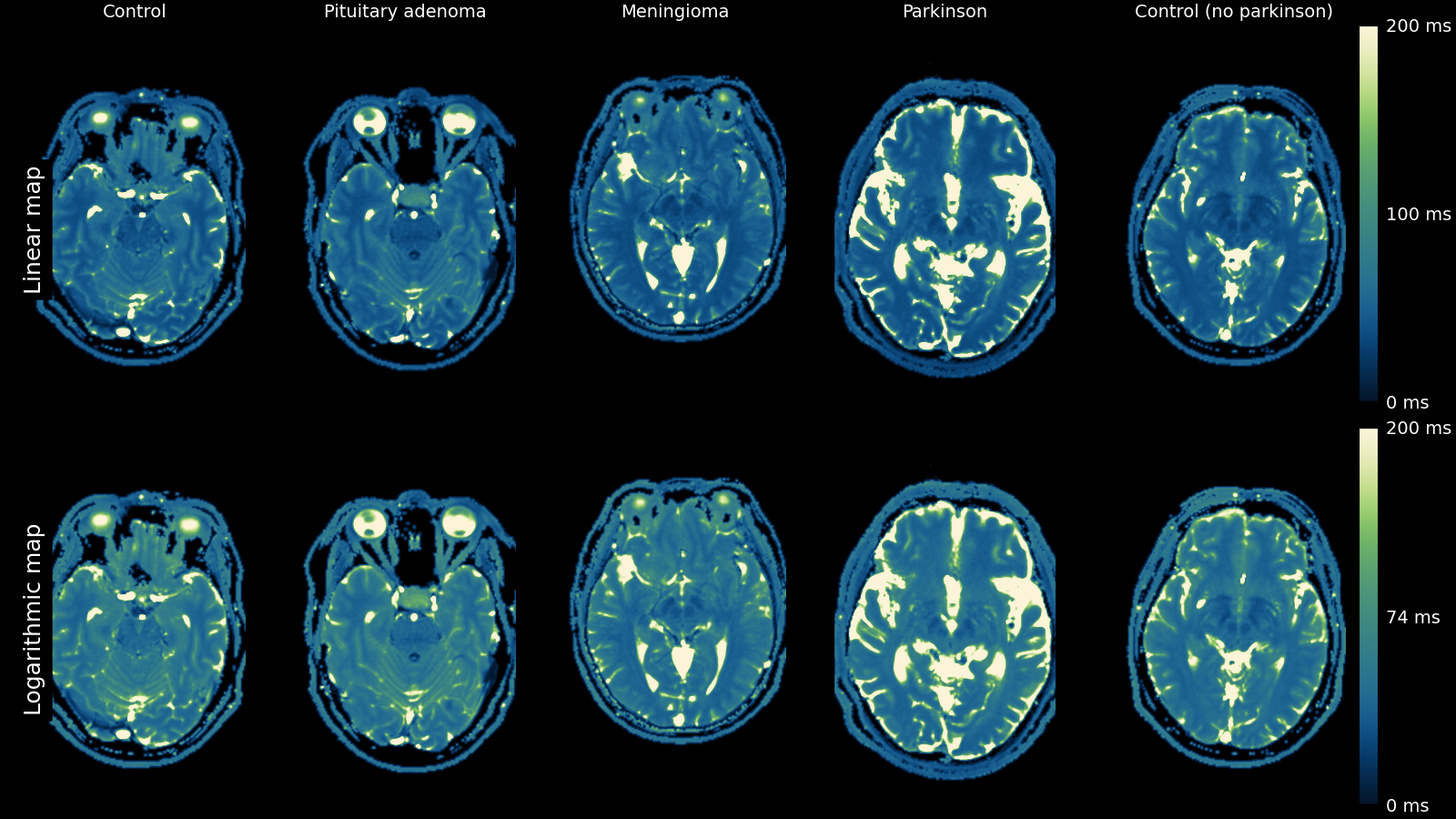


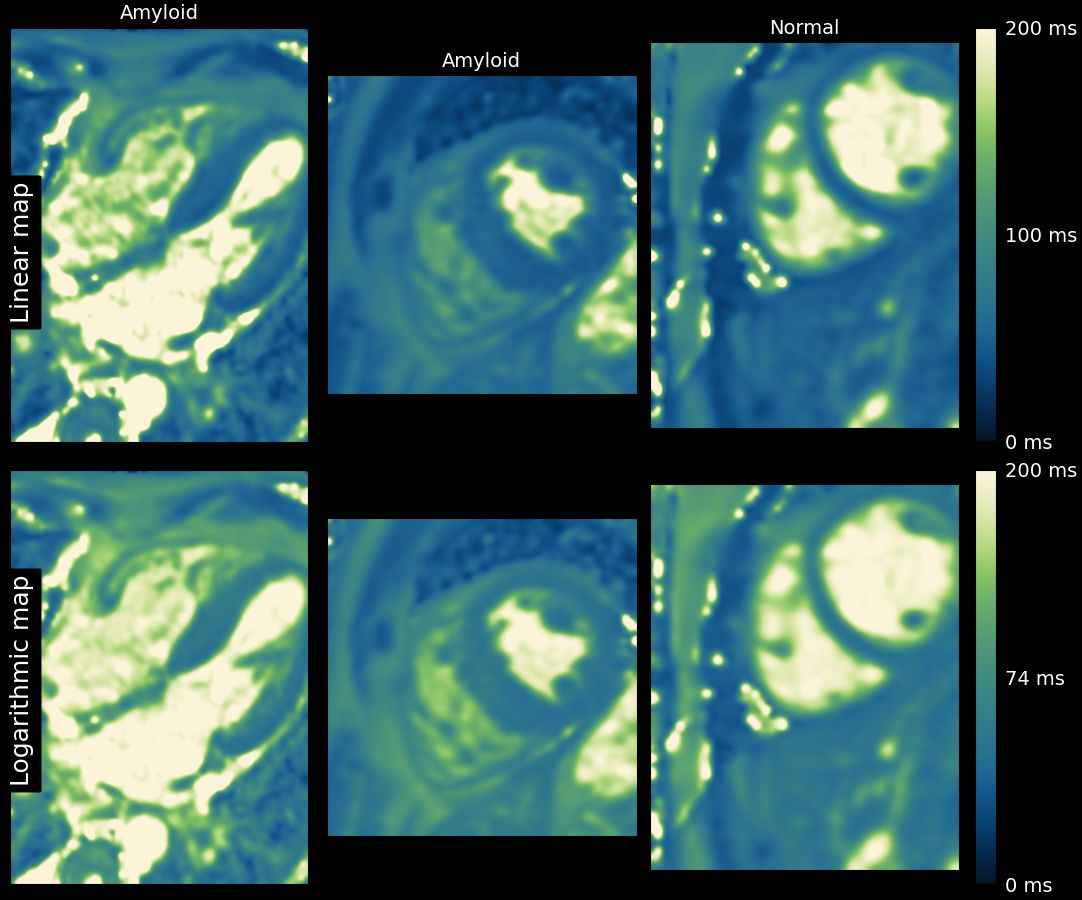


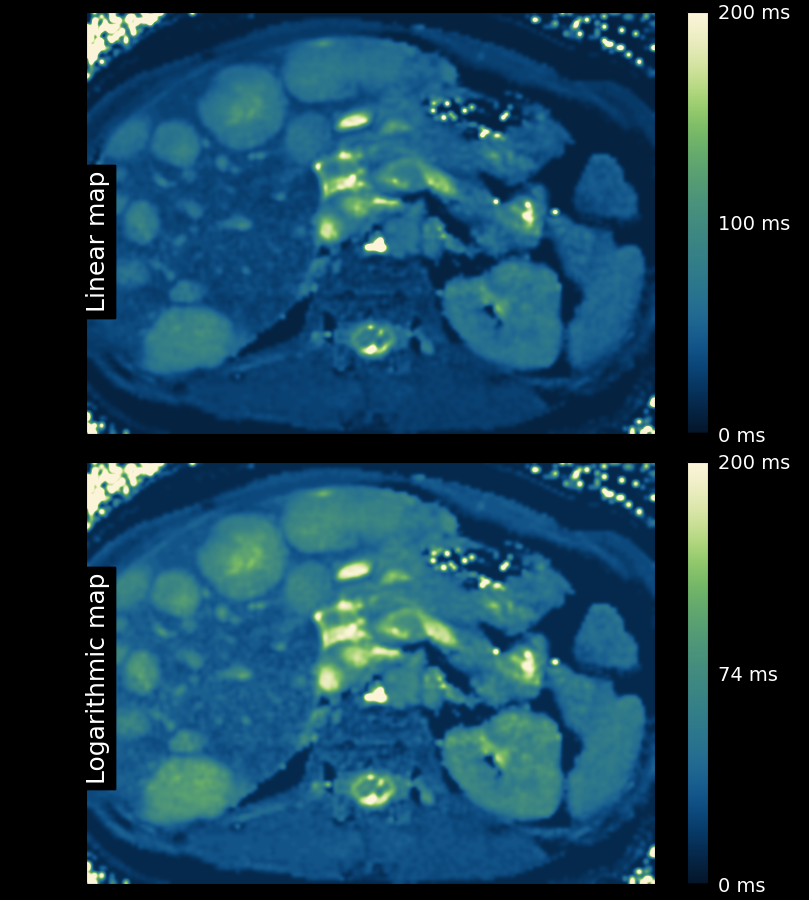


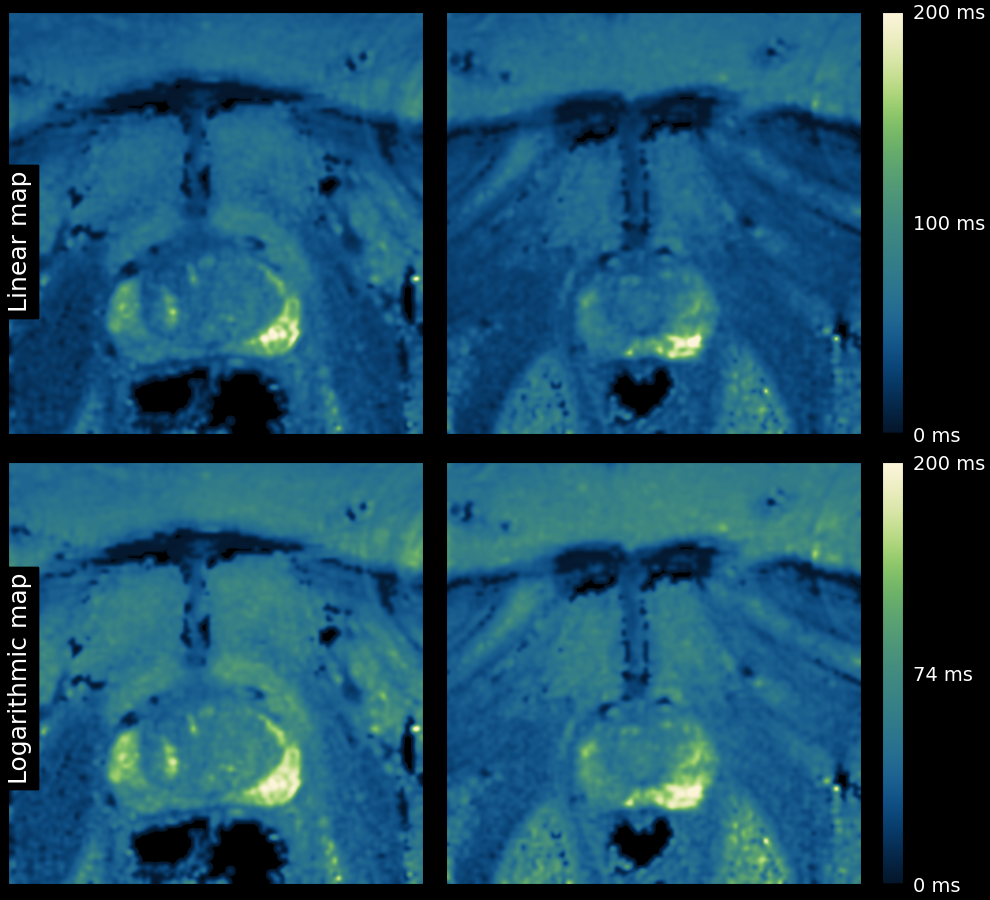


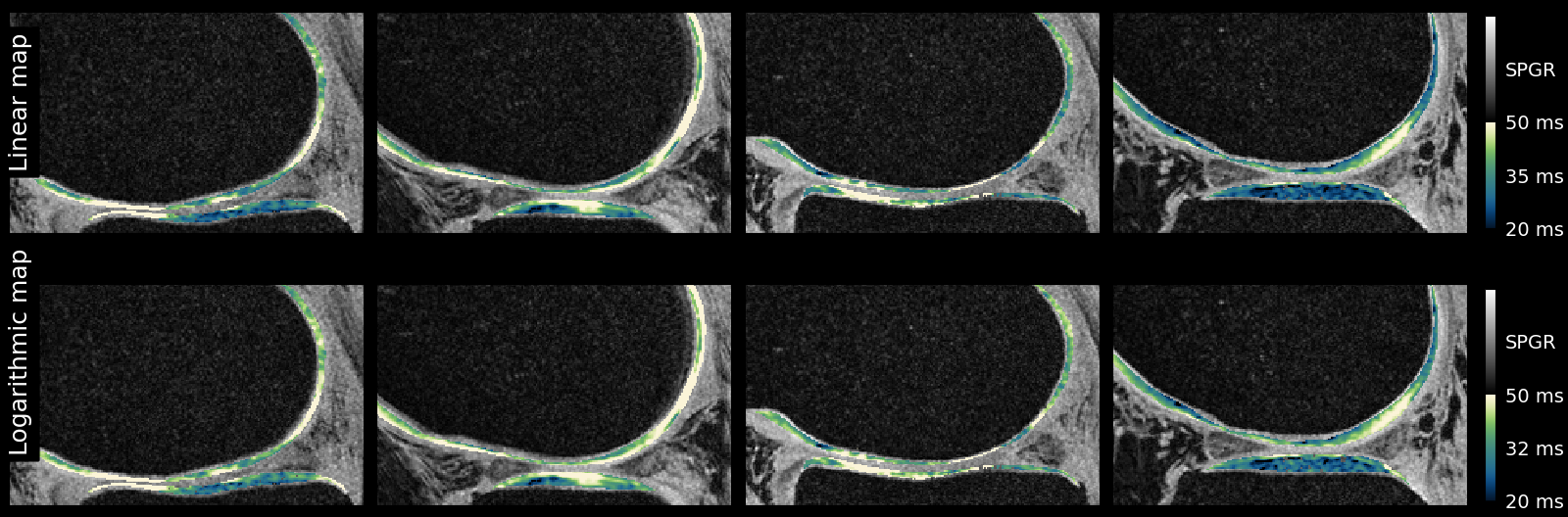

Supplement: Supplementary file 3 — Data S3. The images as presented in the questionnaire of Round 4 of the Delphi process. [file MRM-93-490-s002.docx]
