# Supplementary material for: Color‐map recommendation for MR relaxometry maps
Source: Magn Reson Med. 2024 Oct 16;93(2):490–506. doi: 10.1002/mrm.30290 (PMC11604837; doi:10.1002/mrm.30290)
Supplement: Supplementary file 4 — Data S4. Collection of received comments. [file MRM-93-490-s005.docx]

# Supplementary 4

This material is a dry collection of remarks harvested as part of the Delphi rounds. These have been only very moderately pre-processed for

- Spelling errors
- Interpunctuation
- Removal of plain reiteration of the score (e.g., “I agree”)

In all other regards, this is raw data.

## Round 1

Remarks relating to “(…) same color map for (e.g.) T1 should be used for all anatomies”

- Maps should clearly indicate normal values per organ and per technique. So they could be different for each organ (in terms of absolute value). I would however agree to using a standardized map which clearly indicates mean and SD from normal value for each modality.
- Using different color maps for different anatomies would complicate the interpretation. The specific color should one-to-one relate to the T1 value, not to anatomy.
- To allow direct comparison of the same quantity (e.g. T1) between different anatomies. Think at CSF in the brain and spine, why this should have different colors? CSF value is the same and so it's representation in a figure should be the same. The color map, however, may change for different quantities. One may think that the color map is like a unit of measurement, independent of where this measure is taken.
- Generally, standardized color schemas promote intuitive understanding; already “local” standards w/r/t parametric maps are in use, mostly through analytic software defaults. Promoting intuitive visual communication is therefore useful, but uses should have the option to modify. I also think it’s avoids confusion when using multiple map overlays (e.g., FDG PET +MR maps+ radiation isodose curves).
- T1 has a very high dynamic range of over an order of magnitude from under 100ms to over 2000ms for oedema/fluid. Differences of 10's to 100 ms could be significant but not easily visualized with T1 max of 3000ms for fluid for example.
- Using same map for all anatomies makes it easier to understand color maps across different anatomic region and sites.
- I agree because maybe the range of T1 expected in different organs differs (very narrow range vs broader range) and therefore the ideal colormaps may be differ as well.
- To avoid confusion, the same color scaling for all organs.
- For standardization it is important to have minimal variation in color maps.
- I think a single color map is sufficient, with the limits to be adjustable based on the anatomy.
- Using one colormap for a specific relaxation parameter would be very helpful for readers of journal articles. This ensures that there is limited confusion and easy identification of images while reading scientific papers. One downside may be that there are so many different relaxation parameters that it may get cumbersome to give every parameter its own color scale. In this case, I believe that only a set of the most utilized parameters (e.g. T1 and T2) should have designated colormaps. I am undecided on whether other parameters should have similar or very different options (i.e. R1 could look a lot like T1, but I do not think ADC maps should look like T2 maps).
- There is no reason to use different colour schemes for different anatomies, as long as the boundaries can be adjusted, as the range of values might be different in e.g. the brain from the heart.
- Comparable display of values across body regions and structures.
- Try to be as consistent as possible. Also depends if the scaling of the map remains flexible or fixed to specific values. One could also reserve one contrast to a fixed scaling, one to a moving/flexible scaling.
- When using advanced imaging techniques standardization is often a problem. Consensus not only on acquisition but also on representation of the parametric maps and parameters in the report may be recommendable.
- I see value in standardizing color maps, but worry about range differences between datasets. IE, if a dataset is only demonstrating T1 values over a small range, will it be obvious that the range is being amplified if a standard color map is used. Possibly two standards, a full range standard and a truncated range standard, would be valuable.
- Having different color maps for different anatomies would lead to a zoo of color maps which would be difficult to compare or harmonize.
- Because the color-map is assigned to a parameter instead of being anatomically related
- The range of T1 values for certain types of tissue. I don't think we should squeeze in muscle T1 in the same range as WM and GM. What would be interesting is to have a scale that shows neutral colors for tissue that is in the expected T1 range and stronger for when it gets out of range. As to have a clear distinction.
- Ideally a color scale that is recognizable as T1 would be best, however I can see specific instances where it may be more appropriate to use another color map as the standard color map can't describe the T1 changes in that anatomy appropriately.
- I think there should not be a specific color for a specific anatomy. People may develop unintentional biases because of that, hence standardize a color across all anatomies.
- Different color combinations may be useful to provide a more intuitive representation of different anatomies.
- The colormap should rather point towards a specific parameter (e.g., T1) than towards a specific anatomy.
- I mostly agree, but the ranges covered by the maps should of course be modified to the anatomy and context.

Remarks relating to “How many color maps per anatomy?”

- In essence, we have just variants of TWO fundamentally different properties: longitudinal relaxation (T1, R1) and transverse relaxation (T2, T2*, T1rho and inverses). The color maps of the relaxation rates can be the inverses of the maps for the respective relaxation times.
- We should use one standard map for each time, which clearly shows if the times are normal or abnormal low or abnormal high. They should be the same for all times T1, T2, T2*, T1rho etc.
- Whether or not a single color map is used for all relaxation parameters depends on the user interface: if the relaxation parameter is clearly indicated/printed in the images, one single map may work. If not different maps may be better. But the number of maps may grow if many parameters need to be displayed, which may also not be desired, may be confusing.
- If the unit of measurement is the same (here seconds), then I could argue we should use the same color map. However, it may be helpful to have a set of predefined colors for different relaxation properties. One thing instead should help is the set of limits. For the same anatomy, one should use the same limits, so that visual comparison becomes also easier.
- Aesthetically, I like the idea of spin-spin, spin-lattice and spin-lock “color families” but that’s just my personal preference.
- T1 and T2 variation in tissue is highly non-linear. In fact R1 and R2 maps, in my opinion, are much easier to visualize. for example in brain R1 and R2 maps can visualize variability across all tissue from deep grey to CSF without the need for re-windowing. Although T1 and T2 better relate to standard clinical T1w and T2w images, i think there is an argument for encouraging R1 and R2 maps, color or grey-scale. But if T1 and T2 etc. are to be the main focus, then a linear scale is problematic that would be useful for all anatomies and pathological changes, let alone being still useful but the much compressed ranges of T2* and T2'.
- It depends on the number of possible good colormaps. If that number is limited, then it is better to use fewer instead of e.g. 3 good ones and 1 bad one.
- For consistency and clarity, I go for two maps. The key thing will be to be quantitative for all relaxation times/rates.
- More than two is ideal. But certain combinations of parameter maps are unlikely to be combined in a single study. So it could be possible to produce recommended combinations for the most commonly used/presented metrics.
- In an image, anatomy is more easily distinguishable than the relaxation parameter without any other context. Therefore, I believe the colormap should be different for relaxation parameters and not for anatomy. Furthermore, I research a lot of different anatomy and it would be easier for me to think of T1 as always being one color not dependent on anatomy, and T2 being another color. It would make it much easier to keep track of parameters as I switch between projects. I do not think it would be a good idea to use a specific colormap for every relaxation parameter, because that easily gets to be too much. Perhaps only one or two of the most utilized parameters can have designated colormaps and the others could have a choice of other good options. Related parameters could perhaps share a colormap or a closely related color scheme. I have never seen anyone do inverse colormaps for inversely related parameters like is suggested in the question, but on the surface that makes a lot of mathematical sense. Perhaps this could be done appropriately.
- Probably important to separate the contrasts, so that one starts to automatically associate a particular relaxation time with a color scheme. There could be an interest in reversing the color maps for rates vs. times to keep the message visually consistent (i.e. a short T1 = long R1).
- The amount of color maps has to be limited, yes, this is important, but I think 2 are not enough. I would go for separate ones for T1, T2, T2* and one for all others including T1rho.
- I believe that each of these should have its own colormap: 1. T1 , R1 2. T2, R2 3. T2* , R2* 4. T1 rho, T2 rho (or separate these to two different colormaps).
- Regarding color maps for inverse parameters (T1 vs R1, for example), I worry that using the same colormap is confusing.
- Having a different color map for each relaxation property would make them easily identifiable (i.e. viridis is always T1). Nevertheless, the selection of what is used for T1, T2,... would be completely arbitrary and therefore we should follow the principle "simple is better than complex" and use the same for all parameters.
- Different contrasts should have their own colour map since this is defined across all anatomies
- Don't know what would be best. Not sure about the value of each parameter and how its range should be depicted.
- I have put 5 for the 2nd question as I don't fully understand what it is asking. Ideally a colour map would be defined for 1) T1/R1, 2) T2/R2, 3) T2*/R2*, 4) T1rho/R1rho.
- If there is a fixed color-to-anatomy pairing, I do see unintended consequences (which right now I am not aware of). Also, consensus should not too restrictive, I would not want to miss out on knowledge/listen to someone just because they were not allow to speak (publish) because they not use THE designed color. Sure, there should be guidelines on which color scales are OK to use (based on how humans perceive color/information). How would one compare visually two properties if the colors are different? Sure for quantitative analysis just go with the actual values, but if we don’t care about visual comparison (~qualitative), why the hassle?
- For me, the first two possibilities are conceivable. If there is one perfect colormap, let's use it for all relaxation properties. For me as a physicist it is much more important to read a label (e.g. "T1 / ms") at the color-bar than to replace the need to put one by the choice of the colormap. I think also a splitting in longitudinal and transverse relaxation properties is quite reasonable. However, when it comes to defining one different color map for each and every property, I am wondering if we have enough scientifically useful (and yet pleasing) colormaps available, especially because many studies also include B0 and B1 maps, and maybe other quantifiable properties such as PDFF.
- Using only 2 color maps imposes a type of hierarchy (e.g. T1 is more important than other maps), which might not be the case and might change over time. Using different colormaps would also work, but more properties would require more colormaps while these are not infinite. Using one colormap makes for easy comparison between maps, and gives large flexibility. The obvious downside is the increased chance of human error, if the difference between different properties is not immediately apparent.

Remarks relating to “Should maps be ‘windowable’?”

- The map should be adapted to the normal relative values of the technique, not to absolute values, as they vary by organ and according to the sequence.
- For scientific purpose, I would prefer not to fix the colors to a certain range, but for clinical application this may be desired.
- For a given anatomy it should be fixed. It may vary for different anatomies. Otherwise visual comparison remains still a jungle.
- I would prefer “default” representation be constrained to a fixed value range but allow window & leveling/thresholding post hoc
- Fixed color scales definitely have a role in standardization. But there likely will need to be a range of fixed scales to cover all needs, i.e. different anatomical locations and pathologies.
- This needs consensus.
- Agree. T2 values may be very short or very long. A fixed range can't e.g. reflect the values of an UTE scan.
- I agree as far as these are defined by body parts and by static magnetic field intensity.
- Free adaptability would disregard the organ and field-dependencies of relaxation times.
- I think there should be recommendations for each anatomy (WM vs GM, vs whole brain, vs other body regions etc.). And then authors should have to make a good case as to why they needed to deviate from this colormap.
- I agree slightly with the idea that the range of a colormap should not be fixed. This allows the author to demonstrate subtle contrast differences more easily. In the event that a map shows several values near one another, there would be no way to distinguish anatomy especially when viewed on a screen (or paper) with limited capabilities. Certainly, I want to show very subtle differences between right and left ventricular T1 values. However, this also allows for manipulation of the data as it is presented to overly represent subtle differences. It would be unethical and dishonest to show two patient images in a longitudinal study (e.g. before and after treatment) with different window and level in order to show a more dramatic change than is representative of the data. Allowing a tunable colormap would create a loophole for academic dishonesty. Therefore, I like the idea but not without some restrictions and caution.
- This is absolutely the case. We should be able to adapt the scale as we might lose any contrast otherwise. The aim of a color map is to represent a measurement, which should make sense.
- Flexibility sometimes needed within one color map scheme to be applied to different anatomies.
- I think it will not be possible to cover all used cases with a fixed color map. But I would give a standard as a recommendation.
- I think it would be prudent to have certain maps that are absolute and others that are relative when dynamic range requirements change.
- Range of the color map needs to be adapted to the range of the T1, T2,... values and hence depends on anatomy, field strength, pathology... and cannot be fixed. Nevertheless, recommended ranges for different anatomy and field strength could be defined and authors of publications could be motivated to provide their maps using these ranges either directly in the paper or as supplementary material. This would strongly improve the visual comparability of results presented in papers.
- For example in the case of ex vivo studies, this range varies. Even more, for this kind of choices, will depend predominantly on the histogram of valued pixels. In case of an uniform distribution, then makes sense to fix the scale otherwise needs to be adapted to differentiate better between structures with identical values
- Disagree, Since there can be much variation in the absolute values which are displayed on the map (which actually is the mainstay of detecting pathologies), the range then must be very wide. This in turn would lead to very low contrast in cases without pathologies. Imaging is always about windowing and leveling. So this also should be true for mapping.
- Feel should be fixed for organ/application, but adaptable across body systems to accommodate differences in T1/T2 in different tissues.
- I really like to have this standard. Let's say green is the ok color for that tissue's T1 range. Deviating from that shows higher or lower T1 or other parameter.
- I can see the merit in purple always corresponding to e.g. T2=30 ms; however I feel it would be too restrictive and lead to some applications ending up with a scale that is unable to differentiate anatomical features effectively.
- For standard applications I would support fixed values. for more research oriented tasks it should be flexible.
- Sounds very good to have something like this, but again I’m thinking how is this not going to be gatekeeping.
- In theory, I like the idea of defining fixed ranges per application. In practice, however, I notice that there are discrepancies between relaxation techniques. Some example: while a 2D Multi Echo Spin Echo may show T2 values up to 100 ms in cartilage, a qDESS sequence may show much smaller T2 values, let's say, until 70 ms. How would a fixed scale be able to take that problem into account?
- Most properties have a too large variation over anatomies, while having a smaller range within the anatomy. Fixed ranges would make most of the colormap redundant in many cases.

Remarks relating to the invalid color

“A color map should contain a specific color (e.g. black), clearly distinguishable from all the other colors of the color map, to indicate invalidity or missing data”

- On one hand, it would be very useful to distinguish "invalid" from zero. On the other, it reduces the color range available for the property itself, AND it makes the display pipeline (i.e. software) much more complex.
- Certainly, needed for quality control and misinterpretation.
- I would argue the use of 2 colors: 1 for masked out due to missing data or background, 1 for physics unfeasible data (e.g. negative relaxation times).
- Agreed; null mapped pixels should be readily identifiable as invalid or missing, rather than informative.
- Non-valid data must be represented, it is an indication of image/processing quality (e.g., DKI frequently has issues). Such regions should simply be set to the minimum or maximum. Maximum values are in fact a problem. Pure water at 37C may well have a known accurate T1 (at a guess, about 3500ms). but calculated T1s are likely to range up to 4 or 5sec due to noise - so how will this be represented, or will the data be clipped at the maximum expected?
- Fully agree. To be determined whether black is the ideal color for that.
- This proposal deserves to be considered and delivered further.
- Data intended to be interpreted should not be at the end of the colormap. Therefore, I think it is sufficient to have masked values as one of the limit values.
- I fully agree with this one. There are many situations where the algorithm that produced the map did not run properly on a given pixel due to an artifact or mathematical disruption. I want those pixels to be shown clearly to prevent misunderstandings about data. However, the color chosen may need to be different from background to distinguish it from an unanalyzed region as was stated in Crameri et al.
- This is a good idea. While black is visually very pleasing, a bright or odd colour could also be used to highlight the number of 'failed fit'...
- In my opinion this is highly needed to discriminate data from no data/missing data.
- This may help to place ROI avoiding this potential areas of errors
- Yes, That is because noise. Especially when the scale is too low.
- This really helps in accepting a color scale as it really represents something we need to know, even if it is a faulty value.
- It's important to see “invalid” fits clearly.
- This may not work for dynamic ranges from negative to positive values, e.g. QSM data has negative to positive susceptibility in ppb typically could be, for example, -300 to +300 or above, and 0 ppb has physiological meanings.
- Yes, seems very legit/common sense.
- I think this would increase the interpretability of the data.

Remarks relating to “Quantitative relaxation MR images are always viewed with dark background”

- MRI images and derived color maps are usually viewed in dark environment (radiology reading room). Too bright color maps may not be desired there (similar to not using too bright user interfaces).
- Personally I prefer dark background. However, for printed versions, I think white may be better and environmentally more friendly (less ink to print).
- Traditional radiologic images are displayed with black background; I believe this should the default (reader visual discrimination is higher than against a white field) as a default but should be alterable on-demand by users who want an “inverse background” or fixed gray-scale background.
- I think this needs to be explored by presenting various colormaps with dark to bright backgrounds and assessing what the perceptual changes are.
- This needs consensus whatever is decided should be valid to all scenarios.
- Likely not always.
- This convention may be very hard to break.
- In the past, black backgrounds have always used for all radiology applications (except fluoroscopy). We should not try to change something like this unless there is a clear and justifiable reason. I do not see such a reason for quantitative MR maps like there is for fluoroscopy vs. planar X-ray.
- Radiologists are used to black background, while neuroscientists might have a different opinion. But if we want to have any chance for the radiologists to follow our recommendations, the map need to be on a black background. Only potential exception, the use of white background in quantitative angio, to make MRI looks more like cath angio.
- More common using dark background, I do not see a distinct need to change this.
- Better identification of different colors.
- I generally agree, but wonder about differences in "black" on various screens.
- From personal experience, I would say that a dark background automatically focuses the attention to the bright object of interest. Maybe this also has something to do with the fact, that the black == background, i.e. less important is due to the fact that this is used in the grey value representation of qualitative MR images.
- That is because both the anatomical images (usually in grey scale) and quantitative maps (usually in parula) requires black background for better contrast
- Adaptation of the eyes would be easier and conspicuity of lesions could be easier.
- The prescribed background should be adjusted to give the clearest representation of the results.
- The above example is not convincing in that question, I would not go for it necessarily.
- I would say that a dark background is closest to the standard way of reviewing images in radiology. However, the examples shown here do not use an extra color for invalid data, but white (or black) figures on the color scale. How do we join these two requirements? if we set background as well as missing data to black, we cannot use any scale that already includes black in the color values...
- Not certain if this is corroborated by scientific research, or just because we are more accustomed to it, but I think it helps in interpretability

Remarks relating to “The maps shown in Griswold2018 should serve as a basis (…)”

- For brain imaging I am not sure that the proposed scale for T2 might be appropriate.
- More research and discussion may be needed to reach consensus.
- Very reasonable initial implementation.
- There needs to be a full exploration of (in the first instance) T1 and T2 maps of different anatomical regions and pathologies, a clear understanding of the purpose of the map, e.g. instantaneous appreciation of typical T1, T2 values, or demonstrating subtle pathological changes, and how different color schemes (and axis limits) affect the perception of the information that is presented.
- yes can be starting point would need training.
- I somewhat agree but the font needs to be much larger.
- Not necessarily, but a good starting point. Not sure if cold colors would work for T2?
- The suggested colormap for T2 is perfect (on right, in blue-green), but I disagree with the T1 colormap (left in magenta-red-yellow). In some situations, it is useful to highlight specific anatomy in the image. In the T2 colormap, I can easily show an ROI contour of many different colors on blue-green with excellent contrast for the reader to be able to see. On the T1 map shown, there are not very many contour colors that would have sufficient contrast over magenta/violet, red and yellow making the ROI difficult to distinguish. A cool color contour would be difficult to distinguish in the darker regions, whereas a warm contour (or white) would blend into the higher value regions. Griswold et al. do have the right idea that T1 and T2 should have, in a way, opposing color scales. Perhaps T1 could be a "kry" colormap like shown in Crameri et al. "Kry" is similar but doesn't have the white or magenta/violet as shown in the above map, which I think is the issue.
- Good starting point, but does not have to be these.
- maps on this survey look fuzzy while the numbers on the right are too small to read.
- I particularly think that colormaps pairs like cividis/kry would be preferable to demonstrate differences between colormaps even if colorblindness is present.
- Both colormaps have an increase in brightness from low to high values. In my opinion if something is brighter, the eye automatically attributes more importance to it. Nevertheless, a T1 value of 1000 is not necessarily more important than 500. Having these colormaps would therefore lead to a bias towards higher values. I would therefore suggest to use isoluminant colormaps (as mentioned in Ref [32] of the paper Crameri et al. NatComm 2020: Kovesi, P. Good colour maps: how to design them. CoRR abs/1509.03700 (2015).
- Could be a good start. Not necessarily the best, but a start.
- If these maps have started to become adopted in the literature as recommendations then they should certainly be looked at as the first proposed candidates for T1 and T2 maps.
- We used similar color maps in our paper, but the scale might be different. <https://link.springer.com/article/10.1007/s00723-019-01177-x>
- The notion that 'jet' color should not be used is not novel. I don’t see the very simple argument that people just use(d) jet a lot since it was the default matlab colorscale (for a while, not sure if it is now) or at least a popular one and was used without any misintention per se.
- I don't have a strict preference, but as the maps in Griswold are part of the five (?) existing colormaps that are perceptually homogeneous, chances are quite high that we would adopt them. However, they do include black in the scale, don't they? So they are maybe not the optimal choice if we stick both with a black background requirement and with an invalid pixel requirement.
- Since they are already optimized in various ways, they seem like a very valuable starting point. However, I don't have experience with them yet, which makes me a bit hesitant.

Remarks relating to the importance of perceptual linearity

- Yes, essential requirements.
- Visual intelligibility across color perception range should accommodate most users. Gray scale color mapping will rarely be used practically imho, and is less imperative.
- This really depends on the dynamics of pathology/tissue class changes. T1 and T2 do not change linearly with pathological change, hence a linear scale may reduce the ability to detect subtle changes in short T1 and T2 tissues. This is an area of exploration and assessment.
- Agree with the color blindness linearity, if feasible. I wonder if we should start providing supplementary images with optimized colormaps for color blind people.
- Crameri et al. makes a valid point when showing the nonlinearity of "jet" colormaps: it creates edges where there are none. Further, it needs to be disability accessible despite the limited number of colorblind radiologists and medical physicists. That is an argument that I refuse to give up. It would be ableist to leave those with colorblindness out of this.
- We need to be as inclusive as possible. And it is important for the color map to retain its information when printed on black & white.
- Color maps are already a conversion. Back-conversion does not make sense... Print-outs of papers are not frequently used any longer.
- The color maps should be robust and able to be viewed by any kind of people.
- I think collapsing to greyscale is extremely important as it is a reasonable fallback for all forms of colorblindness.
- Linearity is important but I am not sure this is easy to achieve without having a bias again towards high or low values. For most scientific plots the importance of a value scales with the value but this is not the case for MR mapping, making the design of a color map a very challenging task.
- Of course I like to have full inclusion of people with disabilities, but maybe we should first look into a solution that gives the best outcome for standard situations and see if we then can adapt well to the alternatives.
- Although a fully perceptable colourmap for any kind of color blindness is ideal, this will likely limit the number of options available. There is a vast array of different parameter maps that need to be distinguished in imaging (not just relaxation maps) and it is unlikely that all of these variations can be satisfied for all maps if color blindness viewing is critical. A scale show definitely be clear when converted to greyscale.
- If the goal is to show linearity -> yes colormaps should be linear (if the opposite is the goal, then of course not). The last one is especially important due to some limitations from publishers.
- I think we should try and incorporate the most common color blindness and also the greyscale requirement, as many print publications are just b/w because color printing is expensive. I don’t think we can incorporate any kind of color blindness, or differently speaking, for me it sounds more important that 99.99% of the viewers can benefit from the ideal colormap than using a less ideal one that includes also the 0.01% of the others (especially, if different diseases result in different requirements that cannot be joined in one colormap, which I imagine to be the case).

Remarks relating to the availability for free

- Yes, to accelerate adoption world-wide.
- The consensus color schemas should be made public and available across analytic platforms (and allowed as free-use by vendors).
- Ready availability will certainly encourage their use. but we shouldn’t limit our investigations/proposals to what is currently available if we can demonstrate and justify alternatives. we also need to encourage journals to ask for images to conform to standard scales and perhaps get these as a LUT resource downloadable from the ISMRM/ESMRMB websites.
- Also add Julia, as it is growing rapidly.
- Today's practice.
- These colormaps should absolutely be readily available for free and on commonly used platforms. Otherwise, students and researchers with limited resources would not be able to easily publish their work. That is critically important for the growth of the field and dissemination of research. I would even be willing to code them into Matlab myself and distribute them for free if I have to. Personally, I do not believe you should have to pay for anything other than a cheap admin fee to publish research.
- Pyplot and Matplotlib are primarily for researchers. So it is a secondary aspect. But a licensed color map would make it impossible for people to use and therefore will become hardly enforceable.
- Use of common platforms or standard software may be desirable so that any specialist can use them
- Colormaps should be as accessible as possible (globally).
- Further, code wrappers should be available to make it easy for people to use and a reasonable default should be chosen and set in the various languages.
- If they are not freely available, they are not going to be used.
- If it has a value, it can have price.
- they have to be free and widely adaptable, otherwise how will people use them?
- Colormap script for Pyplot and Matplotlib should be provided to the scientific community as open source and for free.
- I guess the most exact way would be to provide the colors between which the map is defined, wouldn't it? Additionally, one could think of a Github where definitions of the colormap are provided for all Python, Matlab,... versions. I mean, Matlab's viridis could look different from Matplotlib's one, for example. So maybe if users can contribute with code, it will facilitate the use of appropriate colormaps for people who do not like programming.

Additional remarks

- Gray colormap is also important.
- I would strongly suggest maps which clearly indicate if a value is within normal range (i.e., within the normal mean + 2SD) and indicating the degree of abnormality to high or low. Therefor I still like RGB with green normal red abnormal high and blue abnormal low, but the colors could be others so the maps are readable for color deficient people.
- Interesting & important study!
- Exciting effort—great work!
- I think this project is really important. a strongly focused piece of work for just T1, R1 and T2, R2 to cover the whole body and a wide range of field strengths (including the UHF used in animal studies), will be quite a challenge, but set the bar for future studies of other parameters / tissue class and probability maps etc. it could also set methodology on how to develop, justify and get consensus accept for future maps. nevertheless keeping in mind how these parameters relate to T1rho, T2* T2' etc., will be important.
- Quantitative imaging at present is not taught as a rigor in radiology. Its high time quantitative imaging is now brought in tandem as essential for clinical radiology. The training should include the understanding of biases and principles of quantitative imaging.
- I am reiterating here the idea of providing alternate versions of images for colorblind people in the supplementary material. A python/matlab function that creates these images automatically would be very useful.
- Nice job on this first survey. Some of the questions and options were confusing so I anticipate we will need to discuss after the survey is collected.
- The recommendation should be as general as possible for all organs, neuro should not be dominant (despite in practical life it is so).
- Marked differences in parametric values between 1.5T and 3T (and other field strengths) should be explicitly addressed – i.e., if the same scale was applied to a CMR at 1.5T and one at 3T areas of "pathology" would look very different on each.
- Crameri et al. makes plenty of valid points that should be thoroughly adhered to by this initiative. Key takeaways are that colormaps should be disability accessible, linear, uniformly ordered, and easy to read without use of a caption. I believe that while captions are necessary for ultimate clarity, figures and images should be able to tell a clear enough story that the caption does not need to be read. I also think that improvements and standardizations should be made in a manner that does not provide any sort of cover or loophole for specious or unethical behavior (like changing color scales for an overly dramatic representation of subtle data).
- Very good questionnaire. Congratulations to everyone, and especially Miha!
- The question of how many maps should be used is difficult to answer without having distinct examples... But standardizing and limiting them makes full sense.
- I would be happy to provide additional feedback if needed. Good luck with this important initiative!
- Ideally the color maps chosen here for scientific presentations are the same or at least as similar as possible to what is going to be used in clinical practice. Therefore, it would be important to involve clinical experts in this discussion and maybe even carry out a study on a few selected cases to verify that the chosen color map indeed allows for a better (or at least comparable) diagnostic accuracy compared to the state of the art.
- I have concerns that the focus of this should be broader than just relaxation maps. If, for instance, the "best" color scales are taken by T1, T2, T2* and T1rho, we need to consider the implications for representing other parameters, such as ADC, ktrans, ASL perfusion etc
- Very good initiative. I am curious for the second round.

## Round 2

Remarks relating to the set of questions referring to the number of colormaps required

- Whether or not a single color map is used for all relaxation parameters depends on the user interface: if the relaxation parameter is clearly indicated/printed in the images, one single map may work. If not, different maps may be better. But the number of maps may grow if many parameters need to be displayed, which may also not be desired, may be confusing.
- We can design many different scientific color maps, so this should pose no limitation on the number of maps used for relaxometry maps in qMRI.
- T1rho is actually not a scalar tissue property but a continuum: the T1rho(100Hz) will be different from T1rho(1kHz). Does this call for an infinity of color maps? I do not think so. Moreover, what we call “T2” is also a continuum of properties, but in a different range. What we call T2* is the T1rho at 0Hz and what we call “T2” is the T1rho at approximately 20Hz. So we either define an infinity of maps for the collection of {T1rho, T2, T2*} or we define one single map for the collection of the three (possibly, we also include a color map for the dispersion map).
- It would be easier for me to think of T1 as always being one color not dependent on anatomy, and T2 being another color. It would make it much easier to keep track of parameters as I switch between projects
- It has to be clear from a colormap what one is looking at.
- Following some comments of the previous iteration: 1. We are currently only talking about few parameters, but it may become much more complex if we go beyond relaxation with nowhere to end. 2. As such, we should aim at the best possible color map to be used for all quantitative maps but appropriate labeling is to be highlighted.
- It would be great to have distinct color-maps for each physical entity, but at the same time the amount of distinct and distinguishable, nice color-maps is limited, thus a compromise has to be reached. Separate maps for T1, T2 and the rest could be one alternative.
- In general, separate physical constants should be colored differently, but if the underlying physical principles are related (i.e. T2, T2*, T1rho = 'generalized' T2), the same color map might be beneficial to highlight the differences in e.g. sensitivity to a particular physiological change (i.e. T1rho vs. T2 for early stroke). For the last question, on T1 and R1, it should be the inverse, not the same.
- I believe that too many rules and exceptions (e.g. a different colormap for every conceivable parameter, or separate colormaps for colorblind and full vision people) can make it difficult to accomplish this mission effectively.
- Actually, I understand/like both approaches, i.e., "1 colormap recommended for all" and "1 colormap recommended for longitudinal and 1 recommended for transverse relaxation parameters" (and, maybe, 1 recommendation for other quantitative parameters"?). However, I am rather of the opinion that simple is better than too complicated, i.e., I would avoid recommending too many different color schemes).

Remarks relating to “Should maps be ‘windowable’?”

- For the same anatomy, one should use the same limits, so that visual comparison becomes also easier.
- The map should be adapted to the normal relative values of the technique (the bias to the produced maps), not to absolute values, as they vary by anatomy.
- T2 values may be very short or very long. A fixed range can't e.g. reflect the values of an UTE scan.
- There can be much variation in the absolute values which are displayed on the map (which actually is the mainstay of detecting pathologies), the range then must be very wide. This in turn would lead to very low contrast in cases without pathologies. Imaging is always about windowing and leveling. So this also should be true for mapping.
- If we do not fix the ranges, we end up in a jungle of colors. For the same tissue, it will then be impossible to do direct comparison between published work on an image basis as this will appear with different colors. Then at this point what is the value of a standardized color map if we still cannot compare directly the same anatomies. This is even more important for clinical work. Think at follow up exams, you want to maintain the same scale for the same examination, otherwise visual comparison of longitudinal scans are IMPOSSIBLE! The pathology will therefore change colors not based on the response to therapy, but based on the scale that is used. This would be a very BIG issue!
- Very similar to CT where one needs to adjust center and window to highlight specific aspects in subtle greyscale with the rest in black&white.
- There are many reasons to adapt the colormap range such as highlighting artefacts or emphasizing certain tissue types of interest. In addition, defining the range would mean to agree on a range for every relaxation parameter at every possible field strength which sounds like an impossible task.
- Recommended range of the color map should be differ by age of the patient if possible.
- Recommendations must be made, but this should be a strong recommendation and not a "must" for both clinical and research applications. I guess a "must" would limit the adaption rate of users and lead to the usage of completely different settings, including different colors, etc. This contradicts a standardization approach.
- In the clinic, physicians are always windowing and leveling. It makes sense to afford clinicians the ability to do that with quantitative maps. Sometimes, subtle differences in quantitation are difficult to see, but can be critical to diagnosis. In the case of scientific work, the range should be fixed. Allowing the ability scale ranges permits dubious reporting practices. Scaling colormaps is similar to modifying y-axis ranges on boxplots to give the idea that mean differences are more dramatic than they really are. Adjusting colormap scales for the sake of reading an image with better fidelity is acceptable, but reporting quantitative maps should be restricted to prevent nefarious reporting practices.
- Range values may be different according to sequence type, vendor, and MR field strength but also my depend on local variations (remember the recommendation of obtaining the reference values according to the vendor, magnet field strength and type of sequence.
- Be sure to add a color bar.
- Clinical work needs to be reproducible for adoption. The current issue in qMRI is the range of values you can get across scanners, for any given quantitative metric. By forcing a standardized color map, hopefully more work will go into getting repeatable values (thus more accurate measurements). This should make clinical adoption easier. Otherwise, these quantitative metrics are just qualitative!
- For clinical work in the typical CMR service with many different diseases it is good to standardize the results, while for research with some diseases (e.g., with Fabry) it may be worth having a more adaptable approach.
- I think one can recommend ranges per anatomy for clinical work and field strength and maybe also the recommendation to state in a manuscript whenever one is deviating from the recommended ranges - but most probably, one cannot oblige people to always use this exact scale for some reasons that were mentioned in round 1.

Remarks relating to “The maps shown in Griswold2018 should serve as a basis (…)”

- Don’t know - answers from round one leave me in an equally "undecided" position towards this question.
- Not necessarily, but a good starting point. Not sure if cold colors would work for T2?
- The suggested colormap for T2 is perfect (on right, in blue-green), but I disagree with the T1 colormap (left in magenta-red-yellow). In the T2 colormap, I can easily show an ROI contour of many different colors on blue-green with excellent contrast for the reader to be able to see. On the T1 map shown, there are not very many contour colors that would have sufficient contrast over magenta/violet, red and yellow making the ROI difficult to distinguish. A cool color contour would be difficult to distinguish in the darker regions, whereas a warm contour (or white) would blend into the higher value regions.
- I particularly think that colormaps pairs like cividis/kry would be preferable to demonstrate differences between colormaps even if colorblindness is present.
- Both colormaps have an increase in brightness from low to high values. In my opinion if something is brighter, the eye automatically attributes more importance to it. Nevertheless, a T1 value of 1000 is not necessarily more important than 500. Having these colormaps would therefore lead to a bias towards higher values. I would therefore suggest to use isoluminant colormaps (as mentioned in Ref [32] of the paper Crameri et al. NatComm 2020: Kovesi, P. Good colour maps: how to design them. CoRR abs/1509.03700 (2015).
- The colormaps do include black in the scale, don't they? So they are maybe not the optimal choice if we stick both with a black background requirement and with an invalid pixel requirement.
- Since they are already optimized in various ways, they seem like a very valuable starting point. However, I don't have experience with them yet, which makes me a bit hesitant.
- Still needs some serious improvement. E.g. The different blue shades show very little difference between tissue types.
- This is a good starting point but the colormaps should be made more widely available on different platforms (matlab, pytorch) is they are to become a standard.
- My answer has not changed. I think the blue-green colormap for T2 is fine as is, but the warm color based map has issues. The magenta/purple at the lower end of this map does not fit with the rest of the colors in the map. This makes it difficult to determine a color of annotation that demonstrates sufficient contrast from all colors in an image. Even the above image shows a little too much contrast between the pelvic bones and adipose/muscular tissues than what is actually represented (much like the nonlinearity issue with jet colormaps).
- It seems sensible to build upon work that has already been published.

General remarks

- I guess this had already been mentioned at the outset of this journey; it is highly important that industry is on board as for all clinical use, the implementation on their products drives the clinical world.
- I think it important to actually have example T1, R1, T2, R2 etc. maps from brain and body and with pathology at the start of this discussion, as the mapping method and criteria cannot be discussed in isolation of actual data.

## Round 3

Remarks relating to suitability of Lipari

- This looks good. the max value of 2s is less than pure water, about 3 - 4s, and the error in calculating T1 is often high, so apparent T1 can be as high as 4-5s over individual pixels. 2s seems a good compromise so as not to compress the scale - but this will be a number that needs consensus as oedema will be in the range 2s to 3s at 3T.
- This looks good to me, though has a little bit more "range" near the bottom (0.0 to 0.5). I have red-green colorblindness.
- I'm strongly colorblind (due to the lack of red cones). I can see the full extent of the scale.
- It stresses extreme values like from CSF. Clear distinction between GM and WM. However, I'd like to see an example with lesions to see how they appear in relation to GM and WM.
- I like that it has no black voxels within the color map, so one can distinguish between very low values (dark blue) and background.
- The CSF is high T1 structure, the brightest shade allotted to highest T1/CSF makes is distracting to pick lesions The color grading between normal and pathological structures is too narrow (between 1000-1500 ms)
- Does not indicate normal value. might be useful for brain but not for heart, where I would like to have method and sequence specific values.
- Colors are too similar, somewhat greyish.
- This is better than the colormap in Griswold. It's linear, and more understandable at first glance due to the lack of magenta.
- 1. What does the scaling on the side refer to as? The extremes? 2. It would generally be beneficial to present color maps from various organs/organ regions as their usefulness may in fact depend on the 'local' conditions and variations of quantitative biomarkers per organ.
- I would appreciate a bit more saturated colors.

Remarks relating to suitability of Navia

- Nice scale and discrimination of grey and white matter - but pathological data goes much high than 125ms. T2 in CSF can be estimated at 2s or more, in cyst around 500ms - and in oedema 200-300ms. the full T2 range is thus very high and will lead to compression of pathological variation. R2 compresses the cyst-CSF range and so enables a more linear variability of R1 with pathology than does T1.
- I found this colormap harder to differentiate values than the previous one.
- This looks very good to me. Better than the T1 map even.
- I feel other maps (e.g. Lipari) might give a better white/grey matter contrast. Of course, this might only be the case for adult brain T2 maps.
- CSF is clear, but WM and GM have no contrast. Maybe a non-linear scale would be better here as the T2 values for the important tissues are close while CSF is far away. Cysts, peri-vascular spaces etc. may have high values and stand out, but GM and WM now are problematic.
- Same as for T1 - background (black) can be distinguished from low values (blue). Besides, perceptual linearity for all types of vision is a great plus.
- I think it is suitable and it is easier to differentiate from T1 color maps.
- It is visually better than the previous example, but again it is not optimized for pathology quantification, for example a brain tumor with and surrounding perilesional edema will both be hard to discern given that both tumor and edema have high T1 ( > 1000 ms) and there is negligible color transition for values > 1000 ms in this scale.
- Does not indicate normal value. Might be useful for brain, but not for heart.
- Too green - too narrow.
- This is good for the same reason as the T1 map. I like that this T2 map is a complementary color to the T1 map so that it is easily recognizable as different.
- I find the blue-green differences difficult to distinguish.
- Does this Navia colormap allow identification of abnormalities in all organ regions/systems?
- More saturation would be nice; this tends towards greyscale.

Remarks relating to the multiple choice (Lipari/Navia, Lipari/Lipari or Grey/Grey)

- Different color schemes emphasize that there are different ranges for these image types.
- I think the Lipari color map is superior, however I do like the idea of providing two options for the case that authors choose to use different colormaps for different contrasts. I would prefer option 1 if the navia color map were replaced with something like parula
- With red-green colorblindness, honestly the Lipari and Navia are to distinguish. I think the Navia is a little better, and see no big reason not to use 2 maps for those who like that.
- That is a tough decision. In the greyscale, I can see more details in the image, for example, the lighter gray in on both sides of the ventricles. However, I can't tell the scale.
- Since a different range of the Lipari map is used in T1 and T2, I think there are few advantages to using the same map for T1 and T2 per se. However, I feel the white/grey matter contrast in the T2 map is somewhat better using Lipari, which might mean Navia is not the optimal colormap for T2.
- I would favor the first, but I think for many applications, it is good to also have the grayscale. Making both available may be the ideal.
- I'm not directly in favor of the same colormap, but the example showed in 1) definitely is not a good choice for the T2. So if they choice is between only these options, than 2) Lipari for both would be better, even though the scaling is different.
- It is easier to differentiate them and to get an idea of the actual map and number.
- As a clinical radiologist, it helps to have different color maps for T1 and T2.It gives me a sense of what I am looking at, a guesstimate of what values I can expect to get if I draw ROI and makes my workflow easier.
- Should be different than a gray scale image.
- Neither one, T1 and T2 maps could be the same colors, but should need to show normal values. I would prefer Lipari for abnormal high values and Navia for abnormal low values. Grey values should not be used.
- If the colormaps for T1 and T2 are different then it is not necessary to read the caption/annotation. It is abundantly recognizable with no chance for confusion. Having worked with quantitative MRI, I agree that it is somewhat not necessary to have separate colormaps for two different parameters. However, those entering the field or those doing MR research in another field, would have an easier time with two different colormaps rather than one for both. We should cater to those outside of the field for better outreach and research dissemination.
- I prefert Lipari over Navia.
- Although I think different colored colormaps would be ideal and I am happy with the proposed Lipari and Navia, assuming they are available in Matlab, Matlibplot etc. Gray maps for T1 and T2 would be my second preference.
- Should save color maps for other (non-relaxation) parameters.
- 1 We are aiming at color based maps but need to be able to identify different parameters; however, it would be highly important that vendors provide a prominent labeling as otherwise errors may occur in interpretation of clinical color maps potentially resulting in delayed or incorrect patient care.
- There are more quantitative contrasts than T1 and T2, for example susceptibility. I see more of them coming, e.g. iron vs. myelin. I do not think we should reserve / block certain colormaps for T1, T2. Recommending them would essentially do this, if we do not want other maps to be confused with them. While colormaps often show more details, the presented colormaps are quite "shallow" and I do not see less detail in the gray scale version.
- T1 and other maps reflecting the same property (R1, maybe inverse color map, etc..) should have the same color. Same for T2, with another color than T1.
- Because of the similarities between T1 & T2 (see gray maps), a difference in colors should be beneficial in my opinion.
- Different color combinations help to avoid confusion of different data sets (or measurement methods) and make working with both types of data more intuitive/effective.

Remarks relating to “T2 and T2* should get the same colormap”

- T2* has a smaller range than T2.
- They are both transverse contrasts, so it makes sense to share a color map (possibly with different scales).
- If you use the same map, you could easily compare T2 to T2*, which could be useful in some cases. In other cases, using the same map would be just fine.
- As the values are in many cases quite close, the colormap represents values that can be recognized fast then.
- I agree with having same color map, but naturally with different scales.
- I think all maps should get the same color maps, but need to indicate normal values.
- If we have different colormaps for every single quantitative parameter, then it is going to get really chaotic really quickly.
- Better to have a few very good colormaps than a zoo of different maybe less optimal color maps.
- The argument for 'not too many' colourmaps is compelling, although I would prefer different maps for T2 and T2* in an ideal world.
- In case properly labeled I think this is a reasonable approach.
- The same underling physical principles apply in both cases.

Remarks relating to the necessity of a color bar

- I think that each set of images should be displayed with a color bar reference (i.e., it is acceptable to use one color bar for multiple panes that are identically scaled). I generally think that for quantitative comparison, we should "return to the numbers". Comparing work by figures alone ignores the problem of results data not being made available, which is more important for comparison than differing color maps between publications.
- A color bar with numbers may help to understand the findings presented on the quantitative map.
- If the range is not standardized, of course it should be given, otherwise the maps cannot be interpreted. If the range is standardized, it should still be given to indicate the standardized range is used.
- Without a color bar, it is very difficult to evaluate a map.
- And ideally always the same: For cartilage we ideally should come to fixed colors for fixed values. Seeing values deviating would then immediately trigger the attention, while in case of normal values represented by other colors would not trigger the attention. But as was mentioned: maybe too many different views.
- Direct comparison between work should be done explicitly quantitatively, from the data, rather than from a visual representation.
- The color bar should be 1) organ specific and 2) clearly indicate normal values.
- Seems like a good compromise. This effectively prevents concerns that I had about adjusting colormap ranges to more dramatically show insignificant anatomical differences.
- In clinical imaging appropriate diagnosis is the key and this likely depends on the actual values as well as their 'offset' from surrounding 'normal' tissue. And difference between normal and abnormal is likely highly variable across different tissues.
- Such sentence is rather trivial. If a map is presented, also the measurement scale and the unit should be displayed. Otherwise what are we looking at? QuaLITATIVE MRI or quaNTITATIVE MRI? A colormap can also be adaptable a posteriori by clinicians, by changing the ranges, but I strongly believe that if we aim for standardization of colormaps, also their ranges should be standardized for a given tissue, at least recommended for publications to allow direct comparisons!
- Absolutely, a color bar will be necessary to be able to understand the maps.
- Given that a color bar is a scale and fundamental property of the data visualization, I think not having the scale on the image (i.e., avoid a bit of visual clutter) is not reason enough to avoid the possibility to quantify the data (and be sure of the range shown).

Remarks relating to the usefulness of recommended ranges

- Different anatomies and pathologies likely need different ranges - if there were a set of example images with different ranges then i think a consensus could be reached as what range would be appropriate for recommendation.
- Recommendations for ranges in various applications are important. There should be flexibility to modify them when appropriate. I have, for example, done work on very short T2 and T2* and have in some cases modified color maps to clarify differences that would be hard to differentiate on a color map with a very wide range of values.
- I think it is worthwhile to strive towards a standardization, but there might be exceptions. By having (strong) recommendations, people will hopefully minimize deviating from the standard, and argument why they did not follow it if they felt compelled to do so.
- One or two choices may be considered. Adaptation per field strength should be based on the normal and pathological values that are characteristic for that field strength. In the end pathology should be depicted by the same color independent from the field strength. Otherwise, radiologists need to learn double schemes.
- I think recommendations per type of anatomy and field strength are useful, even though I do not expect them to always be followed (sequences with bias on the quantitative values, showing pathology vs. giving an overview on everything,...). Considering the mandatory color bars, however, the need for standardization of ranges becomes a lot less important. So I think recommendations are useful, but not critical.
- Yes, the color bars should be adapted so that the there is a clear abnormal indication corresponds to 95 CI of normal, and then linear to the range of the organ. No point of having a range which does not correspond to the possible values of the organ.
- Recommendations are usually more practical than restrictions. Therefore, I agree with this for the sake of clinical work.
- Need different ranges for brain, knee, myocardium, liver, etc., as the range of clinically normal and abnormal T1/T2s are different.
- I have seen a lot of good argumentation in favor but also against…

General remarks

- This has been an interesting exercise.
- Although T1 and T2 based maps may have different color scales, the adaptability of them (taking into account the necessity of adding the color bar legend with numbers) may help to increase the applicability in clinical practice with the great variety of vendors and sequence schemes applied in each case.
- I would like to see maps of organ systems outside the brain.
- It still is a very difficult topic to address. We might need even more questionnaires, but I think it is also important to keep in mind for what goal we want to use the color: drawing attention to pathology, differentiate anatomical structures, or other. Currently we don't address that in the questions, so people only react from their own work field.
- Thank you for adding example maps, that made answering/deciding much easier!

## Round 4

Remarks relating to suitability of Lipari

- Very difficult to appreciate differences in T1 for cardiac, brain in these examples, prostate too it not that easy. Neither possible to guesstimate what the T1 value could be by looking at the cooler scale nor can I detect focal areas increase/decrease by looking at the maps,
- On the question of color-blindness: I have protanopia. It remains difficult to see small changes, but I'm not sure the map itself is to blame for that.
- I still feel a strong urge to alter the contrast to see the detail.
- Good differentiation, one color range, quite easy to the eye.
- I like the Lipari colormap a lot better than the one shown in pervious iterations. It really shows the scarification on the amyloidosis images well.
- The majority of T1-mapping spectrum is in the more yellow to pink spectrum and it presents a very binary (yellow/pink vs. dark purple) image that is surprisingly very "washed out" appearing.
- I am not sure whether this color maps is suited to show abnormalities well enough - in particular in the cardiac case.
- It is difficult to discern different signals / contrast between tissues.
- I wonder if this is true "Note that in Parkinson, the T1 value of the substantia nigra is higher than in healthy controls." Parkinson's patients are known with reduced neuromelanin content and elevated iron content in the substantia nigra, both lead to a reduced T1 time. Baudrexel NeuroImage 2010 reported both the caudal SN and rostral SN had reduced T1 in the contralateral side in PD patients versus control.
- Presents qT1 in a 'linear fashion', easy to follow.

Remarks relating to suitability of Navia

- It is better than T1 map, but again not ideal for clinical use.
- Even worse for showing increased T2 in amyloid, everything is blue. it is absolutely not acceptable to fix the range of T2 for all modalities. the range should be adapted such that abnormal values (ie T2>50 ms) are clearly visible.
- Water easily pushes the scale to high values, while tissue of interest may have low values. The lower values are therefore more difficult to differentiate. Low cutoff values (clipping) is an option, but not sure if that is always wished for. A color scheme that has stronger differences in color in the lower ranges could be better?
- For T2 usually low values are of clinical interest, e.g. T2~50ms for cardiac imaging. In the above maps, low T2 values appear very dark and are difficult to distinguish. Maybe this is simply a problem of the max/min values of the color bar.
- This map looks very good for the Prostate and Liver MRI, but for the brain and cardiac MRI the whole image looks the same color, and it's difficult to visually appreciate any range of colors.
- Again, intuitively quantitative and 'linear' for T2.
- Note that implicit here is an assumption that noise doesn't have a long T2. But when relaxation parameters are mapped by Fingerprinting acquisitions and reconstructions, sometimes errors in dictionary lookup process leads to pixel noise with long T2 values, which would look like distracting white dots with this map…

Remarks relating to “The logarithmic maps are at least as suitable as linear maps”

- I prefer the logarithmic maps over the linear maps due to improved contrast differences.
- T1 logarithmic maps are better T2 linear maps are better.
- Unsure. In some cases linear seems better (e.g. prostate T1), on others, logarithmic seems better (e.g. liver T2).
- The logarithmic maps are MUCH better.
- I think being able to see relative changes is important, so logarithmic makes sense. It also is quite likely that the error in the fit is larger when the T2 is larger (debatable).
- They "look" nicer and provide greater tissue contrast. However the lack of linearity of scale will make it more difficult to interpret numerically. Hence my positive but slightly mixed rating.
- Logarithmic maps much better differentiate areas in the region of choice than linear ones.
- Same problem, the scale does not allow to visualize the abnormal T1 in amyloid. This is absolutely not helped by a logarithmic scale. The scale should show normal values in one color, and abnormal values in another transition color. This must be modality specific.
- This is not an easy one. Looking at the color distribution only (i.e., the map without the scale bar), I find that the log maps permit better distinction of structures (and it seems also quite intuitive that a 1 ms difference at very low values has the same importance as a difference of 10 ms at higher relaxation time values. However, as far as I see it. we would lose the utility of the scale bar. On a log scale, it is impossible (at least for the average reader) to know the relaxation time values that correspond to the colors in between the color bar labels. E.g. in the upper examples, three relaxation time labels figure next to the color bars (min=dark blue, max=white, and middle value=green). However, it is impossible to know which relaxation times correspond to light blue areas or to light green ones with the log scale. To my eyes, quantitative maps should not lose the connection to numbers, so I am slightly in favor of the linear variant.
- I feel there are many situations where the logarithmic scale enhances the perceived contrast between regions with differences, which could aid in recognizing deviating values. However, a linear map will remain more intuitive, and if a logarithmic map is used it should always clearly be indicated.
- Small changes that may be important will then be emphasized on every scale and that could be very good. However, in data with big standard deviations (non-uniform objects) this may become less favorable.
- I think that linear maps should be preferred since relaxation times confidence intervals/errors must be considered: a symmetric range around the pixel value, and hence the relative color, is easier to visualize with respect to an asymmetric one (also, the reader should be acknowledged of the logarithmic map, since it is more common to find linear maps in displaying parametric maps). On the other hand, logarithmic maps provide brighter maps (there is a bigger percentage of the range of T1s and T2 represented with brighter colors) that help the reader to highlight the morphological information (small contrasts are better seen in brighter maps).
- I actually like the log better than linear in most of these cases. It really adds a lot of readability to the images. The cardiac images show amyloidosis of the myocardium a lot more distinctly. In the linear scale, some of the blood pool tends to be blown out and the log scale circumvents that.
- The logarithmic maps are much more suitable than linear. The linear images fail to capture the subtly of the spectrum for both T1 and T2, and I find the logarithmic is much better for evaluating the spectrum of T2 ranges.
- Your question was unclear so my answer may be wrong. To clarify: either linear or logarithmic is acceptable. Users should be allowed to choose between linear and logarithmic scale based on the pathology or process that needs to be displayed. As long as the scale type (linear or logarithmic), each type is permissible
- Substantial preference for logarithmic map due to theoretical aspect ( similarity T1-R1, T2-R2) as well as noise performance (often approximately relative) and visual perception (mostly in brain images, where the usually irrelevant CSF gets a smaller portion of the color range).
- As a color bar is mandated, I think that having a choice between logarithmic and linear maps would allow more nuanced detail to be shown and allow more adaptability in a given body region/disease. I would anticipate that the a given specialty would converge on a specific map type, i.e. liver imaging on linear and prostate on logarithmic.
- The issue is not 'a big deal', color bar more important than lin/log issue.
- Log maps make more sense to me. Difference in T2 of 2000ms vs. 2020ms is trivial. Difference in T2 between 2ms and 22ms is physically vast.

General remarks

- Both color scales have blue at the lower end of the spectrum. From what I saw in the images, this does help with contrast, but it may be confusing if someone presents an image with low T1 values next to one with low T2 values.
- Not happy with either T1 to T2 maps shown here, hard to get clinical radiologists use these color maps, as the both the quantitative and visual differences are not super evident, not sure how it works for research community. The color ranges need to be optimized.
- I think it is a no-brainer that the logarithmic maps win!
- These scales are completely useless for cardiac imaging. Scales should show normal values and high and low abnormal values clearly. As these values are modality specific, the scales should be modality specific.
- Just want to clarify that I approve the use of logarithmic scales and linear scales. Users should have the ability to choose as long as their choice is explicitly stated and/or is obvious by display of the center value
- In my opinion the last question failed to capture the full range from fully supporting linear to fully supporting log and I would tend to the latter. Otherwise: nice images and looking forward to the publication.
- Looking good for consensus recommendation of color coding.
- Look at how well the methods appear when data is noisy. Many times images are not as perfect as what you show. Need methods that perform well even when there is noise, which can be substantial for accelerated acquisitions.

## Additional small-scale round

(All remarks relating to preference for linear against preference for logarithmic scale)

- Color should be linear to the relaxation parameter. It would be confusing if T1 and T2 color coding would not be uniform.
- I actually prefer letting people choose whatever linearity (Lin or Log) they prefer as along the make the choice obvious. But if I had to choose only one, I would choose Lin because it is simpler. Unless log has a clear and evidence-based advantage (I don’t see it in the samples above), I would vote for simplicity. Always make things as simple as possible, but no simpler, as the Einstein famously said.
- Logarithmic would put too much weight to 'the mid-range' values.
