# Supplementary material for: Color‐map recommendation for MR relaxometry maps
Source: Magn Reson Med. 2024 Oct 16;93(2):490–506. doi: 10.1002/mrm.30290 (PMC11604837; doi:10.1002/mrm.30290)
Supplement: Supplementary file 5 — Data S5. The list of endorsers. [file MRM-93-490-s004.docx]

# Supplementary 5

List of people endorsing the recommendation

- Mohammed Nasir Abubakari, MPhil Medical Physics, University for Development Studies, Tamale -Ghana
- Bradley D. Allen, MD, Northwestern University Feinberg School of Medicine Department of Radiology, Chicago, IL USA
- Eva, Alonso-Ortiz, PhD, Polytechnique Montreal
- Edson Amaro Junior, Associate Professor, University of São Paulo - Brazil
- Rajpaul Attariwala MD PhD FRCPC, AIM Medical Imaging, Vancouver, Canada
- Ryan J. Avery, MD, associate professor of Radiology at Northwestern University.
- Esha (first) Baidya Kayal (last), PhD, Centre for Biomedical Engineering, Indian Institute of Technology Delhi, India
- Thomas Baum, MD, Department of Diagnostic and Interventional Neuroradiology, Klinikum rechts der Isar, Technical University of Munich
- Noam Ben-Eliezer, PhD, Associate Professor, Tel Aviv University, Department of Biomedical engineering
- Olivier BEUF, PhD, INSA Lyon, Université Lyon 1, CNRS, Inserm, CREATIS, UMR5220, U1294, France
- Gabriele Bonanno, PhD. Siemens Healthineers Intl. AG, Bern, Switzerland
- Glenn Cahoon MSc, Olivia Newton-John Cancer & Wellness Centre
- Donnie Cameron, PhD, Department of Medical Imaging, Radboud University Medical Center, Nijmegen, The Netherlands
- John-Paul Carpenter, FESC, Healthpoint Abu Dhabi
- Carlos Andres Castillo Passi, MSc, Pontificia Universidad Catolica de Chile / King's College London
- Sirio Cocozza, MD, PhD - Department of Advanced Biomedical Sciences, University of Naples "Federico II", Naples, Italy
- Gastao Cruz, PhD, Assistant Professor at the Department of Radiology in the University of Michigan
- Provisionally endorsed by: Yves De Deene, Imaging physicist | South Western Sydney Local Health District, Honorary professor of Biomedical Engineering / Medical Physics at Western Sydney University (AU)
- Yanurita Dwihapsari, MSc, Institut Teknologi Sepuluh Nopember (ITS), Indonesia
- Patrick S. Fuchs, PhD, University College London
- Shohei Fujita, MD, PhD, Athinoula A. Martinos Center for Biomedical Imaging, Massachusetts General Hospital
- Clifton D. Fuller, MD, PhD, The University of Texas MD Anderson Cancer Center
- Laura Carretero Gomez, M. Sc., GE HealthCare, Madrid, Spain
- Wolter L. de Graaf, PhD, Canon Medical Systems Europe B.V., Amstelveen, the Netherlands.
- Saumya S Gurbani, MD, PhD, Diagnostic Radiology Resident, Emory University, Atlanta, GA, USA
- Tobias Haueise, M.Sc., Institute of Diabetes Research and Metabolic Diseases, Helmholtz Munich at the University of Tübingen, Tübingen, Germany
- Hye-Young, Heo, PhD, Johns Hopkins University
- Juan A. Hernandez-Tamames, Full Professor, Erasmus MC
- Petra J van Houdt, PhD, department of radiation oncology, the Netherlands Cancer Institute, the Netherlands
- Houchun H. Hu, PhD, University of Colorado
- Harpreet Hyare PhD UCL
- Dimitrios C. Karampinos, PhD from the Technical University of Munich
- Ivan I. Kirov, Ph.D., NYU Langone Health
- Stefan Klein, PhD, Professor at Erasmus MC, Rotterdam
- Dr Sonal Krishan , Medanta hospital , India
- Harald Kugel, PhD, Clinic for Radiology - University of Münster, Münster, Germany
- Liam SP Lawrence, MASc, University of Toronto
- Young Han (First name) Lee (Last name) , MD,PhD ; Yonsei University College of medicine, Severance hospital.
- Tchoyoson Lim
- Carly A Lockard, MS, Carle Clinical Imaging Research Program, Stephens Family Clinical Research Institute, Carle Health, Urbana, Illinois, USA
- Aad van der Lugt, MD, Erasmus MC, University Medical Center Rotterdam, the Netherlands; Chair European Imaging Biomarker Alliance
- Matteo, Maspero, PhD, UMC Utrecht, Imaging & Cancer Divisin, Computational Imaging group, Radiotherapy Department, UMC Utrecht, The Netherlands
- Lucas B McCullum, B.S., The University of Texas MD Anderson Cancer Center
- Bryony L. McGarry, PhD, University of Bristol
- Humberto Monsivais, PhD, The University of Texas at MD Anderson Cancer Center
- Alexander J. Moody, PhD, University of Indiana School of Medicine
- Won-Jin Moon, M.D., Ph.D. Department of Radiology, Konkuk University Medical Center, Konkuk University School of Medicine, SOUTH KOREA
- Maarten Naeyaert, PhD, Vrije Universiteit Brussel (VUB), Universitair Ziekenhuis Brussel (UZ Brussel), Department of Radiology
- Rita, G., Nunes, DPhil, Institute for Systems and Robotics and Department of Bioengineering, Instituto Superior Técnico, Universidade de Lisboa, Portugal
- Edwin H.G. Oei, MD, PhD, Erasmus MC, University Medical Center, Rotterdam, The Netherlands
- Oluwatobi Folorunsho Adeyemi, PhD, University of Abuja
- Allison H. Payne, PhD, Associate Professor of Radiology and Imaging Sciences University of Utah
- Rebecca Rakow-Penner, MD, PhD, Departments of Radiology and Bioengineering, University of California San Diego
- Alexander Rauscher, PhD, University of British Columbia, Department of Pediatrics
- Michael J. van Rijssel, PhD, UMC Utrecht
- Christopher D Rowley, PhD, Assistant Professor, Department of Physics and Astronomy. McMaster University, Hamilton, ON, Canada
- Tales Santini, PhD, University of Pittsburgh, Pittsburgh, PA, USA
- Martin B. Schilder, MSc., University Medical Center Utrecht
- Calder D Sheagren BS, Department of Medical Biophysics, University of Toronto
- Christopher T Sica, Ph.D., Rush University Medical Center
- Dilbag Singh, Ph.D, NYU Grossman School of Medicine, New York, USA
- Marion Smits, MD PhD, Erasmus MC – University Medical Centre Rotterdam, Rotterdam (NL)
- Tom J Syer, MBBS, Department of Radiology University of Cambridge
- David L Thomas; PhD; UCL Queen Square Institute of Neurology, London, UK
- Annette van der Toorn, PhD, Division Imaging and Oncology, UMCU
- Paula, Trujillo, PhD, Department of Neurology, Vanderbilt University Medical Center, Nashville, TN, USA
- Wataru Jomoto, PhD, Hyogo medical university hospital
- Elias Ylä-Herttuala, PhD, A.I. Virtanen Institute, Kuopio, Finland
- Gaoyang Zhao,Ph.D. Candidate,Department of Magnetic Resonance Imaging, The First Affiliated Hospital of Zhengzhou University
